# Supplementary material for: Exploring registered nurses’ perspectives as mentors for newly qualified nurses: a qualitative interview study
Source: BMJ Open. 2024 May 23;14(5):e082940. doi: 10.1136/bmjopen-2023-082940 (PMC11298707; doi:10.1136/bmjopen-2023-082940)
Supplement: Supplementary data [file bmjopen-2023-082940supp001.pdf]

Supplementary file

Interview guide, individual interviews with mentors (translated from Swedish by the first author)

Information:
• Brief oral presentation of the project
• Mentors introduce themselves
• Anonymity and confidentiality
• Information about audio recording and data storage
• Consent to participate in the study
• Test audio recording. Start recording and interview

Interview questions:

1. **Can you tell me what it means to be a mentor?**
    - What made you choose to become a mentor?
    - If you were appointed as a mentor, why do you think you were chosen?
    - What drives you as a mentor? What is your driving force?

2. **Can you tell me about what you specifically focus on as a mentor?**
    - Can you tell me about the organization of mentorship in your workplace?
    - Can you tell me what promotes mentoring in your workplace and what can prevent (inhibit) mentoring in the workplace?
    - How does your work environment contribute to you being able to perform mentoring?
    - Tell me about the preparations to welcome a new colleague

3. **How would you like to be a mentor and develop the mentorship?**
    - Can you tell me what it means to go from being a "helpful colleague" to being a mentor?
    - If you had a mentor who was your role model, can you describe what qualities you liked in his/her mentoring?
    - Tell me how you would develop mentorship in your unit

Final question: Is there anything else you would like to say about mentorship that you don't think I have asked?

General follow-up questions (questions that are adapted during the interview):
- Can you tell me more about/elaborate on ...
- Can you give an example ...
- Do you think that...
