# Supplementary material for: Exploring registered nurses’ perspectives as mentors for newly qualified nurses: a qualitative interview study
Source: BMJ Open. 2024 May 23;14(5):e082940. doi: 10.1136/bmjopen-2023-082940 (PMC11298707; doi:10.1136/bmjopen-2023-082940)
Supplement: Author's manuscript [file bmjopen-2023-082940.draft_revisions.pdf]

BMJ Open is committed to open peer review. As part of this commitment we make the peer review history of every article we publish publicly available.

When an article is published we post the peer reviewers' comments and the authors' responses online. We also post the versions of the paper that were used during peer review. These are the versions that the peer review comments apply to.

The versions of the paper that follow are the versions that were submitted during the peer review process. They are not the versions of record or the final published versions. They should not be cited or distributed as the published version of this manuscript.

BMJ Open is an open access journal and the full, final, typeset and author-corrected version of record of the manuscript is available on our site with no access controls, subscription charges or pay-per-view fees (http://bmjopen.bmj.com).

If you have any questions on BMJ Open's open peer review process please email
info.bmjopen@bmj.com

BMJ Open

Exploring registered nurses' perspectives as mentors for newly qualified nurses: A qualitative interview study

Journal:	*BMJ Open*
Manuscript ID	bmjopen-2023-082940
Article Type:	Original research
Date Submitted by the Author:	07-Dec-2023
Complete List of Authors:	Kallerhult Hermansson, Stina; Umeå Universitet, Nursing<br>Kasén, Anne; Nord universitet, Nursing and Health Sciences<br>Hilli, Yvonne; Nord universitet, Nursing and Health Sciences<br>Vaag, Jonas Rennemo; Nord universitet, Nursing and Health Sciences<br>Norström, Fredrik; Umeå Universitet, Epidemiology and Global Health<br>Bölenius, Karin; Umeå Universitet, Nursing
Keywords:	QUALITATIVE RESEARCH, Nurses, Health Workforce

SCHOLARONE™
Manuscripts

I, the Submitting Author has the right to grant and does grant on behalf of all authors of the Work (as defined in the below author licence), an exclusive licence and/or a non-exclusive licence for contributions from authors who are: i) UK Crown employees; ii) where BMJ has agreed a CC-BY licence shall apply, and/or iii) in accordance with the terms applicable for US Federal Government officers or employees acting as part of their official duties; on a worldwide, perpetual, irrevocable, royalty-free basis to BMJ Publishing Group Ltd ("BMJ") its licensees and where the relevant Journal is co-owned by BMJ to the co-owners of the Journal, to publish the Work in this journal and any other BMJ products and to exploit all rights, as set out in our licence.

The Submitting Author accepts and understands that any supply made under these terms is made by BMJ to the Submitting Author unless you are acting as an employee on behalf of your employer or a postgraduate student of an affiliated institution which is paying any applicable article publishing charge ("APC") for Open Access articles. Where the Submitting Author wishes to make the Work available on an Open Access basis (and intends to pay the relevant APC), the terms of reuse of such Open Access shall be governed by a Creative Commons licence – details of these licences and which Creative Commons licence will apply to this Work are set out in our licence referred to above.

Other than as permitted in any relevant BMJ Author's Self Archiving Policies, I confirm this Work has not been accepted for publication elsewhere, is not being considered for publication elsewhere and does not duplicate material already published. I confirm all authors consent to publication of this Work and authorise the granting of this licence.

For peer review only

Exploring registered nurses' perspectives as mentors for newly qualified nurses: A qualitative interview study

For peer review only

Stina Kallerhult Hermansson,[1] Anne Kasén,[2] Yvonne Hilli,[2] Fredrik Norström,[3] Jonas Rennemo Vaag[2] and Karin Bölenius[1]

[1] Department of Nursing, Umeå University, Umeå 901 87, Sweden.
[2] Faculty of Nursing and Health Sciences, Nord University, Bodø 8049, Norway.
[3] Department of Epidemiology and Global Health, Umeå University, Umeå 901 87, Sweden.

Correspondence should be addressed to Stina Kallerhult Hermansson; stina.hermansson@umu.se

Abstract

Objective: Existing research has focused mostly on mentees' experiences of mentoring rather than mentors' experiences. Therefore, this study describes registered nurses' experiences of being a mentor for newly qualified nurses.
Design: A qualitative interview study based on semi structured individual interviews. Interviews were analyzed using qualitative content analysis.
Participants and setting: A purposive sample of experienced registered nurses (n =21) from health care units in northern Sweden and northern Norway. Inclusion criteria were to have been a mentor to at least one newly qualified nurse, hold permanent employment of 75%–100% as a registered nurse, and to be able to communicate in Swedish or Norwegian.
Results: Our study's findings suggest that being a mentor plays a crucial role in establishing safety in complex work environments. The main theme consists of three themes: feeling motivated in being a mentor; continuously developing the learning environment; and navigating obstacles and cultivating support.
Conclusion: Being a mentor is a complicated role for registered nurses. The mentoring role is beneficial—i.e., positive and rewarding—if facilitated sufficiently in the context of a structured organization. This study brings a more profound understanding of and provides new insights into registered nurses' perspectives and needs regarding being a mentor and the study's findings make an important contribution to the field of nursing regarding the facilitation of mentoring.

Article summary

Strengths and limitations of this study:

- The study employed data triangulation to enhance credibility, included participants of diverse ages, genders, and organizational contexts across municipalities and counties, contributing to a comprehensive understanding.
- Investigator triangulation was a notable strength, involving multiple researchers in the interview process, data analysis, and interpretation, ensuring robustness and reliability in the study's findings.
- The use of an interview guide enabled uniformity in the interview process, enhancing the dependability of the study.
- Reflexivity was addressed in the methods section, in line with the goal of establishing confirmability, ensuring awareness of potential biases and contributing to the study's methodological rigor.
- Transferability was promoted by providing detailed descriptions of the study's context and participants, however, consideration for international perspectives should acknowledge healthcare organizational diversities across different countries as a potential limitation.

Introduction

Mentoring is beneficial and has proven to be vital for the longevity of a registered nurse's working life. It contributes to the retention of registered nurses and supports registered nurses' professional development [1-3]. Nevertheless, implementing mentoring in healthcare organizations is challenging. Literature has shown that a variety of factors contribute to these challenges. For example, there is a lack of standardization and shared views on mentoring in previous research and in clinical practice. High workload, organizational factors, and high staff turnover are other contributing factors [4-6]. The benefits and implications of mentoring are known. However, research is sparse, and little is known about mentors' perspectives. It is reasonable to believe that this knowledge gap contributes to why most mentoring implementations have been short-term and unsuccessful [5]. The present study will explore registered nurses' points of view and their experiences of being a mentor.

Background

The literal meaning of “mentoring” is defined in the Cambridge Dictionary as “the activity of supporting and advising someone with less experience to help them develop in their work” [7]. The actual meaning of mentoring is more complex than the literal definition. Mentoring in nursing can mean many different things, and the concept is used in various ways [8]. Preceptorship and mentorship are often intertwined and are often used synonymously [5, 9]. A mentor can be defined in various ways [10], in the present study, we define a ‘mentor’ as an experienced registered nurse that has been a contact person and support for the newly qualified nurse during their transition and introduction to the workplace. We define a ‘mentee’ as a newly qualified nurse receiving mentoring.

Mentoring can be a dynamic and positive relationship consisting of honesty, close connection, and trust between the mentor and the mentee [11]. Previous studies suggest that from the mentees' point of view, an ideal mentor is a nurse with seniority who is open to communication and feedback; someone who can provide a safe learning environment; and an individual with knowledge, skills, and a positive attitude [12, 13]. Successful mentors are approachable and support professional socialization by promoting a sense of belonging. Mentors counsel, actively listen, and encourage

For peer review only - http://bmjopen.bmj.com/site/about/guidelines.xhtml

development. An effective mentoring relationship in nursing empowers the mentee [12, 13]. Thus, mentoring is needed on several aspects of a new registered nurse's career. Regardless, mentoring programs tends to focus on orientation of the everyday routines and on clinical training [10, 14]. A recent review by Hoover et al. [10] showed that the frequency of mentoring is often up to the mentor and mentee to decide, and the duration can be from three months to more than a year. The mentor-mentee ratio can be anything from 1:1 to 1:5 [10]. However, statistics on mentor to mentee ratios, frequency of contact, duration of mentoring, et cetera, is scarce. An integrative review by Hampton et al. [15] concluded that there is a need for greater standardization of mentoring concepts in nursing. Also, research on the mentors' point of view and what they need to be able to be this ideal kind of mentor, is limited.

The fundamental nature of mentoring consists of multiple support roles and a longer duration than, for example, a traditional introduction, i.e., mentoring can be ongoing with no precise end date, since support is needed continuously [16]. In addition to being a supportive relationship, mentoring can be defined as an organizational intervention to promote the growth and socialization of newly registered nurses in an organization [17]. Although mentoring can be defined in various ways, most studies agree that mentoring is relational and developmental, has career and psychosocial functions, and involves phases and transitions [13]. The phases can range, for example, from discovery and testing to reciprocal interactions; in the end, the mentee perceives themselves as equal to their mentors regarding their professional capability [11].

Dall 'Alba [18] explained this transition to a professional role in terms of becoming. Personal growth and professional development occur in stages that rely on constructive relationships and support from other professionals, e.g., mentors. Becoming a professional is more than obtaining the knowledge and skills from an education. 'Becoming' is a learning process that transforms the whole person while their professional identity is being discovered. Learning professional ways of being involves the integration of knowing, acting, and being the professional in question [18].

In summary, the benefits and implications of mentoring and the qualities of an ideal mentoring relationship are known—from the mentee's perspective. A scoping review by Aldosari et al. [19] showed a lack of qualitative research regarding the experiences and perceptions of mentors in nursing practice. The present study will help fill the knowledge gap of mentoring from the mentors' point of view. The aim of the present study is to describe registered nurses' experiences of being a mentor for newly qualified nurses.

Methods

Design and context

A qualitative study design with individual interviews was chosen to obtain detailed descriptions of the participating mentors' experiences, in line with Brinkmann and Kvale [20]. The composition and reporting of this study were guided The Standards for Reporting Qualitative Research (SRQR) [21]. This study is part of the overarching research program , a comprehensive Nordic project aiming to develop an evidence-based mentorship program to increase support for registered nurses in northern Sweden and northern Norway.

At the time of the interviews, the study participants worked as registered nurses in health care units in hospitals and municipalities in northern Sweden and northern Norway. The healthcare systems in the Nordic countries are organized similarly, although each organizational level has some degree of variation in the content and responsibilities. Hospital care is predominately confined to state and

For peer review only - http://bmjopen.bmj.com/site/about/guidelines.xhtml

For peer review only

county levels, and municipalities are responsible for home care services and elderly and disability care; e.g., nursing homes [22].

Participants and sampling

Participants were selected via purposive sampling [23] in order to find the participants with the best knowledge of the subject matter, i.e., experiences of being a mentor. Since we wanted a range of experiences, the mentors could have various backgrounds, number of mentees, and various mentoring routines in their respective units. First, we sent an email invitation to the operational managers in all units (hospital units and municipal care units) in one northern county in each country. Operational managers from 13 Swedish and 8 Norwegian units approved the invitation. Then, managers investigated interest and provided contact information to registered nurses willing to participate. The inclusion criteria were to have been a mentor to at least one newly qualified nurse, hold permanent employment of 75%–100% as a registered nurse, and to be able to communicate in Swedish or Norwegian. Lastly, we invited 25 registered nurses.

In this stage, four registered nurses in Sweden declined the invitation, and all the invited registered nurses in Norway accepted the invitation. In total, 21 registered nurses participated in the study, from Sweden (n=13), and Norway (n=8). The participants were of various ages, genders, and had a variety of work experiences from different geographic areas and healthcare units in each county. The median age was 43 years (range 31–59). Participants were selected from hospital care units (n =15) that included surgical, medical, neurological, oncology, neonatal, and intensive care units. We also interviewed participants from primary health care and municipal care (n =6).

Data collection

The research team performed one-on-one semi-structured interviews with the participants in January–May 2021 via the digital platforms Teams or Zoom. The participants chose where they were located during the digital interview; it could be at home or at work. We had an agreement with leaders in the participating units that the participants could participate in the interviews during working hours. An interview guide was developed by members of the research team who had experience of being a mentor. The interview guide was inspired by current evidence about mentoring. Reference groups in participating municipalities and counties reviewed the interview guide prior to the interviews, and a pilot interview was performed. The pilot interview confirmed that the interview questions generated answers about experiences of mentoring showing that the interview guide was valid. After consensus was reached in the research team, interview questions were established to be open-ended with reflective elements and focused on mentoring as being and becoming a mentor. Three main questions were asked; “Can you tell me what it means to be a mentor?”, “Can you tell me about what you specifically focus on as a mentor?” and “How would you like to be a mentor and develop the mentorship?”. Each question had follow-up questions, for example, “What made you choose to become a mentor? “. General follow-up questions throughout the interview could be “Can you give an example of...”, “Can you elaborate on…”, et cetera. The median interview time was 59 minutes. Data saturation was deemed sufficient after conducting 21 interviews of varying lengths, with the longest interview lasting 90 minutes. The interviews were perceived as spontaneous and rich in content, and the interviewer followed up on the answers to elicit more detailed narratives. Interviews were audio-recorded and transcribed verbatim.

Data analysis

The data was analyzed using qualitative content analysis, in line with Graneheim et al. [24, 25]. The primary analysis was performed by the first author, SKH with support from AK, and KB. Authors

For peer review only - http://bmjopen.bmj.com/site/about/guidelines.xhtml

started with an overall reading of the text several times. Then a more detailed reading and de-contextualization was carried out. The content of the text was extracted into meaning units, which were condensed to preserve their core meaning. Condensed meaning units were labeled with codes by SKH. SKH sorted and grouped codes with similar features into subthemes. The subthemes with commonalities in content where then abstracted and formulated as themes, see Table 1. The themes were interpreted into one main theme. The analyzing process was non-linear in that SKH went back and forth between the text, the codes, the abstraction, and the writing during the process of analysis. The codes, subthemes, and themes were compared to the original text several times during the analysis to reduce the risk of over-interpretation. This led to adjustments and revising of the 'theme tree' during the process. AK and KB validated the process of analysis, and all authors discussed the themes, sub-themes, and the overall results until consensus was reached.

Table 1. Examples of abstraction process

Condensed meaning units	Codes	Sub-themes	Themes
I would like to be an example; I would like the person sitting next to me to also feel that I am a role model in attitude and care. (I.5. SWE)	Being an example and role model	Striving to be a role model	Feeling motivated in being a mentor
There are always scientific changes, and we have to be receptive to that and I think it is very important that the mentor also has the ability to be flexible. (I.9.NOR)	Flexibility - be open to new knowledge	Facing new challenges	Continuously developing the learning environment
I find it frustrating sometimes. That you don't get that time to sit down and go through things in peace. (I.3.SWE)	Lack of time leads to frustration	Struggling with work organization	Navigating obstacles and cultivating support

Ethical considerations

We followed the recommendations on ethical principles in human research recommendations described in the Declaration of Helsinki [26]. For example, participants were informed orally and in writing about the study, that participation was voluntary, and that they were free to decline to participate at any point during the study prior to publication. Data were handled with confidentiality, i.e., no unauthorized person have access to any data material.

There are always risks involved in interviewing, due to the closeness in interview situations. In other words, there is a risk of the researcher being too close or too distant [27]. Although the interview questions in this study can be regarded as non-controversial, the risk described above was considered during the interviews. In addition, there was also a risk that taking part in the interviews could increase participants' workload, and/or occupy their personal time. This risk was reduced by the previously mentioned agreement with managers that participants were able to complete the interviews during their normal working hours. The risks and the benefits of the study have been taken into consideration, and the benefits have been estimated to outweigh the risks.

Reflexivity

Researchers in this study had no professional commitment to the participating units or prior relationship with any participating registered nurses. SKH, AK, YH, and KB have backgrounds as registered nurses and experiences in mentoring and therefore had a pre-understanding about the issues addressed in this study. They have different specialties and experiences from different fields of

For peer review only - http://bmjopen.bmj.com/site/about/guidelines.xhtml

nursing. The research team also consist of senior researchers in public health and work environments (FN and JRV).

Patient and public involvement

Patients or public were not involved in the design or planning of the study. Reference groups in participating units had the opportunity to give their opinions on the interview guide.

Results

Our study's findings suggest that being a mentor plays a crucial role in establishing safety in complex work environments. The themes and sub-themes are illustrated in Table 2. The main theme reflects participants' experiences of creating safety for their mentees, while contributing to their own professional development, and of developing a learning environment in a complex and stressful work environment. Being a mentor was described as an unclear role that needed to be clarified and defined, however they were still motivated to be a mentor.

Some aspects promoted mentoring, such as experiencing professional development and being a role model, i.e., the first theme, "feeling motivated in being a mentor". The second theme—"continuously developing the learning environment" —covered creating safety while facing challenges and adapting in mentoring. The third theme—"navigating obstacles and cultivating support"—summed up barriers such as lack of time and support from leaders, as well as enabling factors such as supportive colleagues.

Table 2. Overview of results

Sub-themes	Themes	Main theme
Striving to be a role model	Feeling motivated in being a mentor	Being a mentor plays a crucial role in establishing safety in complex work environments
Fostering professional development		
Facing new challenges	Continuously developing the learning environment	
Adapting mentoring based on the mentee's needs		
Struggling with work organization	Navigating obstacles and cultivating support	
Enabling support and collegiality		

The themes and sub-themes are described in more detail in the following sections and verified with quotes from the participants.

Feeling motivated in being a mentor

Participants expressed how they see it as their mission to support the mentee in becoming more secure in their professional role as a registered nurse. Participants were "striving to be a role model"; which they described as one of the most crucial parts of being a mentor. Being a role model was described as multifaceted and involved a combination of personal characteristics and professional competence. Participants also reflected on the reasons why they are mentors. "Fostering professional development" meant that their motivation, interest, and goals promoted being a mentor.

For peer review only - http://bmjopen.bmj.com/site/about/guidelines.xhtml

<u>*Striving to be a role model*</u> was expressed in various ways by the participants; for example, they talked about how they want to show moral support and strived to be trustworthy. They also described that they strived to promote self-confidence in the mentee. The participants wanted to show competence and show that they were available as mentors. As mentors, they also aimed to show a sense of safety to support the mentee in challenging situations. They saw it as exemplary to be transparent and clear about the goals of the mentoring relationship.

The participants described various qualities they strived to have as role models. The experiences they possessed as registered nurses, including communication skills and vast knowledge was part of what made them role models. Having experience was described as valuable in one's capacity to be a mentor. According to the participants, a mentor should be competent to enable sharing of knowledge and create a trusting relationship with the mentee. Having a permissive attitude was described as necessary. Other qualities of a good mentor included sensitivity, patience, and empathy. They desired to be open and neutral as a mentor. According to the participants, a mentor should strive to be self-aware, self-reflective, and convey calmness. Being a role model could consist of different aspects; for example, being there for the mentee, being a source of professional inspiration, and being supportive. One of the participants described "being supportive" as follows:

For me, being a mentor means I'm there to support my new colleagues, to provide a bit more neutrality so that my fellow colleagues can feel safe in talking in general terms about their experiences in their nursing roles, about health and safety, about dealing with colleagues and other professional categories – being somebody that that person can turn to. (Interview 5, SWE)

The participants expressed that they wanted to inspire the mentee to be confident and secure in standing up for their decisions. By continuous mentoring, they saw that the mentee could find the professional competence and motivation to remain in the profession. In addition to showing the mentee how to be confident, participants also wanted to show the mentee that it is permissible to not know everything. It was described as essential not to steer but to guide, a mentor is someone who listens but does not have all the answers, as expressed in the following quote:

Being a mentor means showing you don't have to know everything, but that you can still feel secure in the knowledge that you don't know everything. […] Providing moral support, secure in your professional role to the extent that you can convey a sense of security in your professional role. (Interview 1, SWE)

<u>*Fostering professional development*</u> meant that the participants were personally interested in supporting a new colleague. Being a mentor was part of the profession; they described it as 'coming with the territory'. Participants expressed that they appreciated the positive response they got from the mentees. When the mentee was interested in learning, participants were encouraged. The participants saw it as rewarding that mentoring promoted their own professional development. Growing in the profession was described as promoting job satisfaction. Mentoring created security and joy at work for both the mentor and the mentee, according to the participants. The participants expressed feelings of excitement in being a mentor. They felt they had the capacity and talent to be suitable to be a mentor and had a lot of experience and knowledge to offer. Mentors were sometimes selected by management, which could be flattering and motivating but could also jeopardize their interest and motivation as a mentor. The goal of being a mentor was to facilitate competent colleagues and ensure the professional development of the department, and mentoring could lead to the development of a sustainable work environment. Participants described it as 'quality assurance'. This participant shared how professional development in mentoring could benefit patient care:

For peer review only - http://bmjopen.bmj.com/site/about/guidelines.xhtml

But sharing the knowledge born of experience is precisely what's so important if others are to succeed in their work, and also to achieve the best possible patient outcomes. So, I think exchanging experience is incredibly important. (Interview 15, NOR)

Regardless of how they became a mentor, the participants described factors that guided them in their mentoring. Being a mentor contributed to their professional development. Seeing mentees thrive in the profession was described as 'rewarding' and increased participant's own competence. Participants described that their competence was made visible during mentorship. A motivational factor in being a mentor was that some participants did not have a mentor when they themselves were new nurses, and they described that they missed having a mentor. In contrast, others described the drive to give the same support they had received as new nurses. Being interested in mentoring was essential to be a successful mentor, as described by this participant:

But then I think most people could do a good job as mentors if they have an interest in it and the desire to do it. But I think you have to have an interest in it – that's the top priority. To be able to do a good job, you have to want to do it. (Interview 6, SWE)

Continuously developing the learning environment

The participants described fast-changing healthcare, which places high demands on a learning environment that support staffs in continuous development. The participants described how new nurses, in their opinion, needed different support today than they did a few years ago. "Facing new challenges" meant that they, as mentors, needed to keep up with new and updated knowledge. The participants reflected that there was no 'best way' to be a mentor, they had to "adapt their mentoring based on the mentee's needs" and knowledge base. A mentor was thought to be essential, regardless of other introductory efforts and routines. The mentees' individual needs guided how long the mentorship lasted and the content of the mentoring.

<u>*Facing new challenges*</u> meant that as mentors, they needed to have "humility for the new", as described by the participants. Mentoring and support were needed not only in clinical situations but also in interactions with other professional categories. The participants tried to foster a permissive atmosphere, where mentees felt safe asking questions. The participants also saw a need for mentoring for all new nurses, but it could be challenging if the new nurse did not think they needed mentoring. It was also described as challenging not to 'give too much' but to encourage the mentee to develop their own abilities.

Participants saw talking about feelings as essential, and that the most challenging aspect was dealing with existential issues if the mentee, for example, had cared for a dying patient. There were challenges keeping up with the development of the current requirements placed on new nurses. The participants saw a need for a longer mentoring period than simply an introduction. They had to be receptive to new knowledge, as described by this participant:

You feel you keep your wits about you when you're a mentor, that you constantly have to go on learning new things. And not least, you have to be sure that what you're teaching is correct and up to date on a professional level. (Interview 8, NOR)

<u>*Adapting mentoring based on mentee's needs*</u> meant that mentoring could vary based on the mentee's previous experiences. With a confident mentee, the mentor could be less 'strict'. However, with an insecure mentee, the mentor had to be more hands-on, according to the participants, and guide the mentee more regularly. Mentoring could be adapted to quick and irregular check-ins, as described by this participant:

For peer review only - http://bmjopen.bmj.com/site/about/guidelines.xhtml

Perhaps dealing with mentoring "on the hoof", so to speak, there and then, but then maybe also being able to sit down and give it time. You adapt to the situation. (Interview 17, SWE)

Several participants described a matching process for the mentee–mentor relationship, where they saw how personal chemistry affected the mentoring. The participants emphasized they needed to know the mentee to plan the mentorship, not only the mentee's previous work experience, but also family life, other obligations outside working hours, etc.

A mentoring conversation stimulates reflection and consideration, according to the participants. The participants tried to focus on the positive aspects of the mentee's development. The ambition was to let the mentee control what was addressed in the discussions, based on the mentees' individual needs. The participants encouraged the mentee to prepare for the mentoring conversation. To be a mentor was also described by the participants as to guide in conflicts, for example if there was a disagreement with a colleague. To continuously follow up on challenges in the job.

The mentoring conversations were based on confidentiality to enable openness. The participants expressed that the aim was to meet one another unconditionally. They thought it was better with freer frames and flexibility around the conversation. It could be anywhere, based on the conditions in the workplace, if it were in a trustworthy environment. For example, mentoring could be adapted into a short and spontaneous reflection after a work shift. Mentoring can develop the mentees' professional skills without resorting to criticism, according to the participants.

But it depends entirely on who I'm mentoring, [the mentee] is the one who should decide what we're going to talk about. (Interview 2, SWE)

Navigating obstacles and cultivating support

The participants expressed experiences of navigating obstacles in the work environment, that could hinder being a mentor fully. They described "struggles with work organizations", and they experienced pressure from leaders and colleagues. The participants also reflected on factors that cultivated mentorship in a supportive environment. They reported that mentoring was enabled if leaders were attentive, and the organization was well-functioning. "Enabling support and collegiality" was also expressed as a necessary part of a supportive environment. Participants also claimed that having structure and predictability in the working day helped them in planning mentoring activities, and that structure and predictability were essential elements of cultivating a supportive environment.

Struggling with work organization meant that participants expressed obstacles related to and frustration with how leaders and management handled mentorship. Responsiveness and sensitivity of managers were lacking, according to the participants. Participants had experienced that they were handed responsibility but not power to influence. Participants requested clarification regarding what mentorship was supposed to be, to be able to navigate the mentor role. For example, this participant expressed thoughts of ending their mentorship due to unclear definitions of the role:

Given that [the mentoring role] has been a bit woolly, I was almost starting to think I should give up mentoring because I have plenty of other things going on at work. [...] Maybe things are getting a bit much, and if I had to turn anything down, then the mentoring would be the first thing to go. (Interview 7, SWE)

They said it was not beneficial for the work environment to 'be forced' to be a mentor. Participants also perceived mentoring to be a non-priority in the eyes of management. Mentoring was often de-prioritized for other tasks in the workplace.

For peer review only - http://bmjopen.bmj.com/site/about/guidelines.xhtml

It was challenging to be a mentor due to insufficient training in, for example, conversational techniques and motivational conversations. They expressed that "mentoring in mentoring" and contact with other mentors were lacking. The will was there—but education and development in mentoring needed to be improved to be able to cultivate a supportive environment. The participants described experiences of staff shortages and high staff turnover that led to obstacles in mentoring. For example, participants had experiences of simultaneously mentoring several new nurses, which could lead to difficulties in navigating the time management and priorities.

They expressed a need for better planning and structure within the organization. It was described as necessary with sensitivity between the workgroup and the mentee. However, there needed to be a more precise division of responsibilities for follow-up in the mentoring. They experienced pressure from colleagues and felt responsible for the mentee and their skills. It was described as challenging to mentor someone whom colleagues saw as an insecure mentee.

Difficulties in planning, such as scheduling factors, could be obstacles for mentoring; there needed to be more time set aside for mentoring. The lack of time led to frustration, and not having set aside time made learning difficult, according to the participants. They needed help preparing. For example, they prepared for mentoring outside working hours. They streamlined mentoring due to lack of time, which led to insufficient development in the mentee. The participants experienced difficulties in navigating mentoring activities during periods of high workload. However, they saw a greater need for mentoring during these periods. This frustration is expressed by one of the participants in this quote:

The management must take the time to follow up. No progress will be made unless they do. But they often feel there's no time for it, so it's not a priority. So, it's there, but it grinds to a halt. (Interview 14, NOR)

<u>*Enabling support and collegiality*</u> meant that a structured environment was essential for being a mentor. Receiving support from team leaders reduced the obstacles and improved the possibilities to enable mentorship. Good management and leadership led to personalized mentoring, according to the participants. When leaders made it possible for them to navigate in both informal and formal structures for mentorship, this was an example of an attentive leader. This is expressed in the following quote:

I also have to make it clear that we have a good manager who pays attention to everyone and understands everyone. She's also really good at keeping things on track. I've often felt that as our manager, she's the glue that bonds the group together. (Interview 12, NOR)

The participants stated that flexibility in scheduling and planning was a prerequisite for being a successful mentor. According to the participants, having time allocated for both mentoring and patient-related work was a prerequisite for cultivating support. Having time for reflection (less workload) also promoted being a mentor. The participants described that having a mentee could reduce but also increase the workload. They described alternating mentorship as preferable, having some space between mentorships and replacing each other during absences.

The participants expressed that it was important to be loyal to one another, even though they were all different and could do things differently. It was described as essential to receive support from other mentors (for example, to talk about obstacles and "difficult cases"). Sharing the responsibility and supporting other mentors and colleagues were considered cultivating. Participants expressed a will to build a value base for mentoring, create a mentorship structure, navigate a plan together.

For peer review only - http://bmjopen.bmj.com/site/about/guidelines.xhtml

They gave examples of mentor groups where they planned new and reflected on previous mentorships. They expressed that they needed more regular meetings to exchange experiences.

They also shared how everyone in the workgroup took part in the mentoring, but only some had the title ’mentor’. Discussions were requested between their colleagues in the workgroup on frameworks and responsibilities regarding mentorship. Participants expressed the importance of taking care of one another. According to the participants, everyone in the workgroup had to be prepared and welcoming to the mentee, and the social work environment was essential for the mentees' learning. The workplace had to be inclusive and responsive, according to the participants. Participants also expressed that supportive leaders and a supportive organization were essential for them continuing to be a mentor. Attitudes and organizational culture were described as vital components in successful mentoring:

But just like here, people need a management team that does the same thing. I can’t be the only one, I rely on us having the same ideas about [mentoring]. It’s really important. (Interview 13, NOR)

Discussion

The principal findings are that the complexity of the mentor role was evident in the registered nurses' narratives, and they strived to establish safety for mentees in complex work environments. Results show that the mentor role lacked clarity and entailed positive as well as negative experiences. For example, participants talked about being motivated, continuously developing a learning environment, and navigating obstacles and cultivating support.

Strengths and weaknesses of the study

Methodological considerations are based on Lincoln and Guba's evaluative criteria on establishing trustworthiness [28]. Credibility was achieved through data triangulation, as participants were selected to encompass a variety of ages and genders, as well as differences in organizations/healthcare units across participating municipalities and counties. This approach included participants with diverse experiences, thereby enhancing the credibility of this study. Another strength of the study was investigator triangulation, i.e., peer-debriefing, which means that multiple researchers conducted the interviews, analyzed the data, and interpreted the findings. We used the same interview guide to enable the interviews to be as uniform as possible to strengthen the study's dependability. We accounted for reflexivity under the methods section, therefore sought to establish confirmability, as described by Lincoln and Guba [28]. Transferability was established by giving sufficient descriptions of the study’s context and participants. The study's transferability to an international perspective should, however, be carried out in consideration of the diversities of healthcare organizations in different countries.

A limitation of the study can be that the first author (SKH) analyzed the interviews but did not conduct any of the interviews. It can be a disadvantage where part of the latent content, such as nonverbal behaviors and interactions, can be missed in the analysis. Having a variety of backgrounds and understandings in the research team broadened the discussions and proved an advantage in our ability to understand the data. We have described our pre-understanding, and our diversity of backgrounds is also a way to prevent bias and achieve consensus through co-creation; as different researchers in a study can interpret the data differently [24]. The researchers speak Swedish and Norwegian, and our variety in native languages was a strength, e.g., we could validate the transcripts and analysis by ensuring that nothing got lost in translation.

For peer review only - http://bmjopen.bmj.com/site/about/guidelines.xhtml

Feeling motivated being in the role of a mentor

The results of the study show that being a mentor includes emphasis on creating trusting relationships with their mentees and being able to share knowledge with one's mentees. These results are in accordance with previous studies that concluded that mentoring success depends on the quality of the mentoring relationships, i.e., the personal connection between the mentor and the mentee [29-31]. Also, according to Dall 'Alba [18], people cannot become professionals by themselves; they can fully develop through contact and integration with others. Participants in our study strived to be a role model and offer support to their new colleagues, while at the same time develop in their own new professional identity of being a mentor.

In addition to supporting their mentees' professional development, being a mentor was also described as beneficial for the participants' own personal and professional development, as confirmed in previous research [30]. The participants in our study expressed that being a role model, being trustworthy, and having competence and experience as a nurse were prerequisites for mentoring. This confirms previous descriptions of an ideal mentor from the mentees' point of view that emphasized similar characteristics [12, 13].

Continuously developing the learning environment

The present study adds new perspectives to previous studies regarding mentorship. For example, a systematic review by Kakyo et al. [30] concluded that the content of mentoring is vital for it to work. In contrast, our results showed that it was important to have freer frames and adapt mentoring based on mentees' needs. Our results showed that content was not the most important aspect of mentorship; the participants in our study emphasized more about *how* and *why* mentoring was performed in their narratives. To develop a learning environment, a need to build a value base for mentoring and create a mentorship structure together with other mentors and colleagues was shown.

The experiences of developing the learning environment and being a role model can be compared to how Dall 'Alba [18] describes "learning to become a professional" as an interplay between "what I am and what I can become". This means inheriting a world of other professionals, as well as inventing one's own world as a professional. Our study shows similar findings. Being a mentor meant having a sense of security in their own professional role and instilling self-confidence in their new colleagues, so that they, in turn, can build their own professional identities.

Other than getting to know their mentee and adapting to their personal learning needs, participants in the present study also expressed challenges in keeping up with the rapid development of new knowledge. A meta-synthesis by Mlambo et al. [32] supports these findings by concluding that organizations should adequately make continuing professional development accessible for registered nurses to keep the staff up to date.

Navigating obstacles and cultivating support

The results of the present study show experiences of staff shortage and time shortage, which hindered participants' ability to perform mentoring activities and participate fully in being a mentor. The participants expressed a need for education, training, meeting other mentors, and the importance of relationships, i.e., 'not doing it alone'. These results are confirmed by previous research that showed how education, training, and time allocated for the mentor to interact with the mentee are prerequisites for organizations to benefit from mentoring [30]. Other studies showed that workload management and scheduling influence how mentoring can be conducted and showed

For peer review only - http://bmjopen.bmj.com/site/about/guidelines.xhtml

that nurse managers expressed similar barriers to mentoring, such as competing priorities for time, and lack of training [29, 33].

Understaffing is related to emotional exhaustion and lower well-being in the workgroup. The higher workload from understaffing also leads to role ambiguity; i.e., staff being unclear about their roles [34]. Results in our study confirms findings of role ambiguity, as participants described thoughts of ending the mentorship due to unclear definitions of the responsibility it entailed and their role as mentors. In addition, participants in the present study also described other challenges; needing structure, i.e., clear leadership and direction, to optimize the mentoring. These findings can be interpreted as participants experiencing inequity, that their input in mentorship is unequal to the outcomes they receive, as described in *equity theory* by Adams [35]. Inequity can, according to Adams [35] lead to frustration, job-dissatisfaction, and a person leaving their position.

The results of our study both confirmed and expanded prior research. Even so, mentoring is still not a part of everyday routines in all healthcare units. The importance and benefits of mentoring are known, but it is still not facilitated sufficiently. According to Dall 'Alba [18], a person's possibilities to act and be are constrained by specific situations, as well as their history, traditions, and conditions. The current situation in health care is not ideal for fully developing mentoring. There is a vicious cycle, where the high turnover of registered nurses leads to difficulties in implementing mentoring that contributes to difficulties retaining registered nurses. Reports on the global shortage of registered nurses have concluded that organizations must create positive work environments that maximize registered nurses' health, safety, and well-being, and improve or sustain their motivation to prevent further shortages. Registered nurses are attracted to work and remain working due to, for example, opportunities to develop professionally, gain autonomy, and participate in decision-making [36, 37].

Moreover, the results of the present study show the frustration evident in the participating registered nurses' narratives. They did not feel valued by the organization and experienced inequity. Therefore, they could not thoroughly add value to their mentorships or to the organization in return. A systematic review of interventions that promote registered nurses' retention by Lartey et al. [38] concluded that most interventions have focused on retaining newly qualified nurses. However, keeping experienced registered nurses in the profession is essential for providing high-quality care. Organizations need to invest in experienced registered nurses to encourage them to remain in the profession, to provide high-quality care, and as mentors for new nurses [6]. Therefore, listening to the registered nurses' experiences and giving them opportunities to develop professionally and participate in decision-making about mentoring are essential factors in keeping them in the profession and for their well-being.

For peer review only

Conclusions

Being a mentor is a complicated role for registered nurses. The mentoring role is beneficial, i.e., positive and rewarding, if facilitated effectively. The role and responsibilities in the mentoring relationship need to be fully established and defined. A structured organization is essential to promote mentoring, as is taking experienced registered nurses' views into consideration. The structure of mentoring in nursing should be designed and facilitated in co-creation with experienced registered nurses as mentors.

For peer review only - http://bmjopen.bmj.com/site/about/guidelines.xhtml

Clinical implications

This study brings a more profound understanding of registered nurses' perspectives of being a mentor. This study offers evidence as to how mentoring should be facilitated, thus contributing to highly competent registered nurses and safer patient care.

Suggestions for further research

More research is required to fully explore and implement mentoring as a concept. In addition, more research is needed to develop, test, and evaluate evidence-based mentorship programs in healthcare units and to create sustainable mentoring solutions.

Declarations

Ethics approval and consent: The Swedish Ethical Review Authority (Dnr 2020-06187) and the Norwegian Centre for Research Data (No. 148896) have approved the study. Written informed consent for participation and publication was obtained.

Data sharing statement: No data are available.

Competing interests: None declared.

Funding: This work was supported by Nordland County [20/1879-14].

Authors contributions:
SKH: Data analysis, Writing – original draft, Writing – review & editing.
AK: Data collection, Validation of analysis, Writing – review & editing.
YH: Project administration, Supervision, Data collection, Writing – review & editing.
FN: Supervision, Writing – review & editing.
JRV: Data collection, Writing – review & editing.
KB: Project administration, Supervision, Data collection, Validation of analysis, Writing – review & editing.

Acknowledgements: Our deepest gratitude to all the participating registered nurses for generously sharing their narratives. We want to thank Johan Åhlin and Jonas Carlström for collecting data. We also want to thank Nord University and Umeå University for the support they provided, making it possible to carry out the study.

References

1. Pham TTL, Teng CI, Friesner D, Li K, Wu WE, Liao YN, Chang YT, Chu TL: **The impact of mentor–mentee rapport on nurses' professional turnover intention: Perspectives of social capital theory and social cognitive career theory**. *Journal of clinical nursing* 2019, **28**(13-14):2669-2680.
2. Schroyer CC, Zellers R, Abraham S: **Increasing Registered Nurse Retention Using Mentors in Critical Care Services**. *The health care manager* 2020, **39**(2):85-99.
3. Mijares AH, Radovich P: **Structured Mentorship and the Nursing Clinical Ladder**. *Clinical Nurse Specialist* 2020, **34**(6):276-281.
4. Coventry TH, Maslin-Prothero SE, Smith G: **Organizational impact of nurse supply and workload on nurses continuing professional development opportunities: an integrative review**. *Journal of Advanced Nursing* 2015, **71**(12):2715-2727.

For peer review only - http://bmjopen.bmj.com/site/about/guidelines.xhtml

5. Edwards D, Hawker C, Carrier J, Rees C: **A systematic review of the effectiveness of strategies and interventions to improve the transition from student to newly qualified nurse**. *International journal of nursing studies* 2015, **52**(7):1254-1268.
6. Jangland E, Gunningberg L, Nyholm L: **A mentoring programme to meet newly graduated nurses' needs and give senior nurses a new career opportunity: A multiple-case study**. *Nurse education in practice* 2021, **57**:103233-103233.
7. **Cambridge Dictionary** [https://dictionary.cambridge.org/dictionary/english/mentoring]
8. Olorunfemi O: **Mentoring in Nursing: A Concept Analysis**. *International journal of caring sciences* 2019, **12**(1):142-148.
9. Zhang Y, Qian Y, Wu J, Wen F, Zhang Y: **The effectiveness and implementation of mentoring program for newly graduated nurses: A systematic review**. *Nurse Educ Today* 2016, **37**:136-144.
10. Hoover J, Koon AD, Rosser EN, Rao KD: **Mentoring the working nurse: a scoping review**. *Human resources for health* 2020, **18**(1):52-52.
11. Hale RL, Phillips CA: **Mentoring up: A grounded theory of nurse-to-nurse mentoring**. *J Clin Nurs* 2019, **28**(1-2):159-172.
12. Chen CM, Lou MF: **The effectiveness and application of mentorship programmes for recently registered nurses: a systematic review**. *J Nurs Manag* 2014, **22**(4):433-442.
13. Mullen CA, Klimaitis CC: **Defining mentoring: a literature review of issues, types, and applications**. *Annals of the New York Academy of Sciences* 2021, **1483**(1):19-35.
14. Gularte-Rinaldo J, Baumgardner R, Tilton T, Brailoff V: **Mentorship ReSPeCT Study: A Nurse Mentorship Program's Impact on Transition to Practice and Decision to Remain in Nursing for Newly Graduated Nurses**. *Nurse Lead* 2023, **21**(2):262-267.
15. Hampton KB, Smeltzer SC, Ross JG: **Evaluating the transition from nursing student to practicing nurse: An integrative review**. *Journal of professional nursing* 2020, **36**(6):551-559.
16. Hansen J: **Results from the national council of state boards of nursing's transition-to-practice study, part 2**. *Journal for nurses in professional development* 2015, **31**(4):240-241.
17. Krofft K, Stuart W: **Implementing a Mentorship Program for New Nurses During a Pandemic**. *Nursing Administration Quarterly* 2021, **45**(2):152-158.
18. Dall'Alba G: **Learning Professional Ways of Being: Ambiguities of becoming**. *Educational Philosophy and Theory* 2013, **41**(1):34-45.
19. Aldosari N, Pryjmachuk S, Cooke H: **Newly qualified nurses' transition from learning to doing: A scoping review**. *International journal of nursing studies* 2021, **113**:103792-103792.
20. Brinkmann S, Kvale S: **Doing interviews**, 2nd edition. edn. London: SAGE Publications Ltd; 2018.
21. O'Brien BC, Harris IB, Beckman TJ, Reed DA, Cook DA: **Standards for Reporting Qualitative Research: A Synthesis of Recommendations**. *Academic Medicine* 2014, **89**(9):1245-1251.
22. **The Nordic Medico-Statistical Committee and The Nordic Social Statistical Committee; Nordic Health & Welfare Statistics** [https://nhwstat.org/health/organization-health-services]
23. Creswell JW: **Qualitative inquiry and research design : choosing among five approaches**, 3., [updated] edn. Thousand Oaks: SAGE Publications; 2013.
24. Graneheim UH, Lindgren BM, Lundman B: **Methodological challenges in qualitative content analysis: A discussion paper**. *Nurse Educ Today* 2017, **56**:29-34.
25. Graneheim UH, Lundman B: **Qualitative content analysis in nursing research: concepts, procedures and measures to achieve trustworthiness**. *Nurse Educ Today* 2004, **24**(2):105-112.
26. **World Medical Association Declaration of Helsinki: Ethical Principles for Medical Research Involving Human Subjects**. *JAMA : the journal of the American Medical Association* 2013, **310**(20):2191-2194.
27. Haahr A, Norlyk A, Hall EO: **Ethical challenges embedded in qualitative research interviews with close relatives**. *Nurs Ethics* 2014, **21**(1):6-15.

For peer review only - http://bmjopen.bmj.com/site/about/guidelines.xhtml

28. Lincoln YS, Guba EG, Pilotta JJ: **Naturalistic inquiry**. *International journal of intercultural relations* 1985, **9**(4):438-439.
29. Devey Burry R, Stacey D, Backman C, Donia MBL, Lalonde M: **Exploring pairing of new graduate nurses with mentors: An interpretive descriptive study**. *Journal of clinical nursing* 2020, **29**(15-16):2897-2906.
30. Kakyo TA, Xiao LD, Chamberlain D: **Benefits and challenges for hospital nurses engaged in formal mentoring programs: A systematic integrated review**. *Int Nurs Rev* 2022, **69**(2):229-238.
31. Lin J, Chew YR, Toh YP, Radha Krishna LK: **Mentoring in Nursing: An Integrative Review of Commentaries, Editorials, and Perspectives Papers**. *Nurse Educ* 2018, **43**(1):E1-E5.
32. Mlambo M, Silén C, McGrath C: **Lifelong learning and nurses' continuing professional development, a metasynthesis of the literature**. *BMC nursing* 2021, **20**(1):62-62.
33. Merga MK, Hays A-M, Coventry T: **Nurse managers' perceptions of barriers to the mentoring of early career nurses**. *Mentoring & tutoring* 2020, **28**(1):60-77.
34. Hudson CK, Shen W: **Consequences of work group manpower and expertise understaffing: A multilevel approach**. *Journal of Occupational Health Psychology* 2018, **23**(1):85.
35. Adams JS: **INEQUITY IN SOCIAL-EXCHANGE**. *Advances in experimental social psychology* 1965, **2**(4):267-299.
36. Buchan J: **Global nursing shortages**. *BMJ : British medical journal* 2002, **324**(7340):751-752.
37. Drennan VM, Ross F: **Global nurse shortages: the facts, the impact and action for change**. *British medical bulletin* 2019, **130**(1):25-37.
38. Lartey S, Cummings G, Profetto-McGrath J: **Interventions that promote retention of experienced registered nurses in health care settings: a systematic review**. *Journal of nursing management* 2014, **22**(8):1027-1041.

For peer review only

For peer review only - http://bmjopen.bmj.com/site/about/guidelines.xhtml

Standards for Reporting Qualitative Research (SRQR)*

http://www.equator-network.org/reporting-guidelines/srqr/

Page/line no(s).

Title and abstract

**Title** - Concise description of the nature and topic of the study Identifying the study as qualitative or indicating the approach (e.g., ethnography, grounded theory) or data collection methods (e.g., interview, focus group) is recommended	
**Abstract** - Summary of key elements of the study using the abstract format of the intended publication; typically includes background, purpose, methods, results, and conclusions	

Introduction

**Problem formulation** - Description and significance of the problem/phenomenon studied; review of relevant theory and empirical work; problem statement	
**Purpose or research question** - Purpose of the study and specific objectives or questions	

Methods

**Qualitative approach and research paradigm** - Qualitative approach (e.g., ethnography, grounded theory, case study, phenomenology, narrative research) and guiding theory if appropriate; identifying the research paradigm (e.g., postpositivist, constructivist/ interpretivist) is also recommended; rationale**	
**Researcher characteristics and reflexivity** - Researchers' characteristics that may influence the research, including personal attributes, qualifications/experience, relationship with participants, assumptions, and/or presuppositions; potential or actual interaction between researchers' characteristics and the research questions, approach, methods, results, and/or transferability	
**Context** - Setting/site and salient contextual factors; rationale**	
**Sampling strategy** - How and why research participants, documents, or events were selected; criteria for deciding when no further sampling was necessary (e.g., sampling saturation); rationale**	
**Ethical issues pertaining to human subjects** - Documentation of approval by an appropriate ethics review board and participant consent, or explanation for lack thereof; other confidentiality and data security issues	
**Data collection methods** - Types of data collected; details of data collection procedures including (as appropriate) start and stop dates of data collection and analysis, iterative process, triangulation of sources/methods, and modification of procedures in response to evolving study findings; rationale**	

For peer review only

For peer review only - http://bmjopen.bmj.com/site/about/guidelines.xhtml

**Data collection instruments and technologies** - Description of instruments (e.g., interview guides, questionnaires) and devices (e.g., audio recorders) used for data collection; if/how the instrument(s) changed over the course of the study	
**Units of study** - Number and relevant characteristics of participants, documents, or events included in the study; level of participation (could be reported in results)	
**Data processing** - Methods for processing data prior to and during analysis, including transcription, data entry, data management and security, verification of data integrity, data coding, and anonymization/de-identification of excerpts	
**Data analysis** - Process by which inferences, themes, etc., were identified and developed, including the researchers involved in data analysis; usually references a specific paradigm or approach; rationale**	
**Techniques to enhance trustworthiness** - Techniques to enhance trustworthiness and credibility of data analysis (e.g., member checking, audit trail, triangulation); rationale**	

Results/findings

**Synthesis and interpretation** - Main findings (e.g., interpretations, inferences, and themes); might include development of a theory or model, or integration with prior research or theory	
**Links to empirical data** - Evidence (e.g., quotes, field notes, text excerpts, photographs) to substantiate analytic findings	

Discussion

**Integration with prior work, implications, transferability, and contribution(s) to the field** - Short summary of main findings; explanation of how findings and conclusions connect to, support, elaborate on, or challenge conclusions of earlier scholarship; discussion of scope of application/generalizability; identification of unique contribution(s) to scholarship in a discipline or field	
**Limitations** - Trustworthiness and limitations of findings	

Other

**Conflicts of interest** - Potential sources of influence or perceived influence on study conduct and conclusions; how these were managed	
**Funding** - Sources of funding and other support; role of funders in data collection, interpretation, and reporting	

*The authors created the SRQR by searching the literature to identify guidelines, reporting standards, and critical appraisal criteria for qualitative research; reviewing the reference lists of retrieved sources; and contacting experts to gain feedback. The SRQR aims to improve the transparency of all aspects of qualitative research by providing clear standards for reporting qualitative research.

For peer review only

For peer review only - http://bmjopen.bmj.com/site/about/guidelines.xhtml

**The rationale should briefly discuss the justification for choosing that theory, approach, method, or technique rather than other options available, the assumptions and limitations implicit in those choices, and how those choices influence study conclusions and transferability. As appropriate, the rationale for several items might be discussed together.

<u>**Reference:**</u>

O'Brien BC, Harris IB, Beckman TJ, Reed DA, Cook DA. **Standards for reporting qualitative research: a synthesis of recommendations.** *Academic Medicine*, Vol. 89, No. 9 / Sept 2014 DOI: 10.1097/ACM.0000000000000388

For peer review only

For peer review only - http://bmjopen.bmj.com/site/about/guidelines.xhtml

BMJ Open

EXPLORING REGISTERED NURSES' PERSPECTIVES AS MENTORS FOR NEWLY QUALIFIED NURSES: A QUALITATIVE INTERVIEW STUDY

Journal:	*BMJ Open*
Manuscript ID	bmjopen-2023-082940.R1
Article Type:	Original research
Date Submitted by the Author:	26-Apr-2024
Complete List of Authors:	Kallerhult Hermansson, Stina; Umeå Universitet, Nursing<br>Kasén, Anne; Nord universitet, Nursing and Health Sciences<br>Hilli, Yvonne; Nord universitet, Nursing and Health Sciences<br>Vaag, Jonas Rennemo; Nord universitet, Nursing and Health Sciences<br>Norström, Fredrik; Umeå Universitet, Epidemiology and Global Health<br>Bölenius, Karin; Umeå Universitet, Nursing
**Primary Subject Heading**:	Nursing
Secondary Subject Heading:	Nursing
Keywords:	QUALITATIVE RESEARCH, Nurses, Health Workforce

SCHOLARONE™
Manuscripts

I, the Submitting Author has the right to grant and does grant on behalf of all authors of the Work (as defined in the below author licence), an exclusive licence and/or a non-exclusive licence for contributions from authors who are: i) UK Crown employees; ii) where BMJ has agreed a CC-BY licence shall apply, and/or iii) in accordance with the terms applicable for US Federal Government officers or employees acting as part of their official duties; on a worldwide, perpetual, irrevocable, royalty-free basis to BMJ Publishing Group Ltd ("BMJ") its licensees and where the relevant Journal is co-owned by BMJ to the co-owners of the Journal, to publish the Work in this journal and any other BMJ products and to exploit all rights, as set out in our licence.

The Submitting Author accepts and understands that any supply made under these terms is made by BMJ to the Submitting Author unless you are acting as an employee on behalf of your employer or a postgraduate student of an affiliated institution which is paying any applicable article publishing charge ("APC") for Open Access articles. Where the Submitting Author wishes to make the Work available on an Open Access basis (and intends to pay the relevant APC), the terms of reuse of such Open Access shall be governed by a Creative Commons licence – details of these licences and which Creative Commons licence will apply to this Work are set out in our licence referred to above.

Other than as permitted in any relevant BMJ Author's Self Archiving Policies, I confirm this Work has not been accepted for publication elsewhere, is not being considered for publication elsewhere and does not duplicate material already published. I confirm all authors consent to publication of this Work and authorise the granting of this licence.

For peer review only

EXPLORING REGISTERED NURSES' PERSPECTIVES AS MENTORS FOR NEWLY QUALIFIED NURSES: A QUALITATIVE INTERVIEW STUDY

Stina Kallerhult Hermansson,[1] Anne Kasén,[2] Yvonne Hilli,[2] Fredrik Norström,[3] Jonas Rennemo Vaag[2] and Karin Bölenius[1]

[1] Department of Nursing, Umeå University, Umeå 901 87, Sweden.
[2] Faculty of Nursing and Health Sciences, Nord University, Bodø 8049, Norway.
[3] Department of Epidemiology and Global Health, Umeå University, Umeå 901 87, Sweden.

Correspondence should be addressed to Stina Kallerhult Hermansson; stina.hermansson@umu.se

ABSTRACT

Objective: Existing research has focused mostly on mentees' experiences of mentoring rather than mentors' experiences. Therefore, this study describes registered nurses' experiences of being a mentor for newly qualified nurses.
Design: A qualitative interview study based on semi structured individual interviews. Interviews were analyzed using qualitative content analysis.
Participants and setting: A purposive sample of experienced registered nurses (n =21) from health care units in northern Sweden and northern Norway. Inclusion criteria were to have been a mentor to at least one newly qualified nurse, hold permanent employment of 75%–100% as a registered nurse, and to be able to communicate in Swedish or Norwegian.
Results: Our study's findings suggest that being a mentor plays a crucial role in establishing safety in complex work environments. The main theme consists of three themes: feeling motivated in being a mentor; continuously developing the learning environment; and navigating obstacles and cultivating support.
Conclusion: Being a mentor is a complicated role for registered nurses. The mentoring role is beneficial—i.e., positive and rewarding—if facilitated sufficiently in the context of a structured organization. This study brings a more profound understanding of and provides new insights into registered nurses' perspectives and needs regarding being a mentor and the study's findings make an important contribution to the field of nursing regarding the facilitation of mentoring.

ARTICLE SUMMARY

Strengths and limitations of this study:

- The study employed data triangulation to enhance credibility, included participants of diverse ages, genders, and organizational contexts across municipalities and counties, contributing to a comprehensive understanding.
- Investigator triangulation was a notable strength, involving multiple researchers in the interview process, data analysis, and interpretation, ensuring robustness and reliability in the study's findings.
- The use of an interview guide enabled uniformity in the interview process, enhancing the dependability of the study.
- Reflexivity was addressed in the methods section, in line with the goal of establishing confirmability, ensuring awareness of potential biases and contributing to the study's methodological rigor.
- Transferability was promoted by providing detailed descriptions of the study's context and participants, however, consideration for international perspectives should acknowledge healthcare organizational diversities across different countries as a potential limitation.

For peer review only

Begränsad delning

INTRODUCTION

Mentoring is beneficial and has proven to be vital for the longevity of a registered nurse's working life. It contributes to the retention of registered nurses and supports registered nurses' professional development (1, 2, 3). Nevertheless, implementing mentoring in healthcare organizations is challenging. Literature has shown that a variety of factors contribute to these challenges. For example, there is a lack of standardization and shared views on mentoring in previous research and in clinical practice. High workload, organizational factors, and high staff turnover are other contributing factors (4, 5, 6). The benefits and implications of mentoring are known. However, research is sparse, and little is known about mentors' perspectives. It is reasonable to believe that this knowledge gap contributes to why most mentoring implementations have been short-term and unsuccessful (5).

The literal meaning of "mentoring" is defined in the Cambridge Dictionary as "the activity of supporting and advising someone with less experience to help them develop in their work" (7). The actual meaning of mentoring is more complex than the literal definition. Mentoring in nursing can mean many different things, and the concept is used in various ways (8). Preceptorship and mentorship are often intertwined and are often used synonymously (5, 9). A mentor can be defined in various ways (10), in the present study, we define a 'mentor' as an experienced registered nurse that has been a contact person and support for the newly qualified nurse during their transition and introduction to the workplace. We define a 'mentee' as a newly qualified nurse receiving mentoring.

Mentoring can be a dynamic and positive relationship consisting of honesty, close connection, and trust between the mentor and the mentee (11). Previous studies suggest that from the mentees' point of view, an ideal mentor is a nurse with seniority who is open to communication and feedback; someone who can provide a safe learning environment; and an individual with knowledge, skills, and a positive attitude (12, 13). Successful mentors are approachable and support professional socialization by promoting a sense of belonging. Mentors counsel, actively listen, and encourage development. An effective mentoring relationship in nursing empowers the mentee (12, 13). Thus, mentoring is needed on several aspects of a new registered nurse's career. Regardless, mentoring programs tends to focus on orientation of the everyday routines and on clinical training (10, 14). A recent review by Hoover et al. (10) showed that the frequency of mentoring is often up to the mentor and mentee to decide, and the duration can be from three months to more than a year. The mentor-mentee ratio can be anything from 1:1 to 1:5 (10). However, statistics on mentor to mentee ratios, frequency of contact, duration of mentoring, et cetera, is scarce. An integrative review by Hampton et al. (15) concluded that there is a need for greater standardization of mentoring concepts in nursing. Also, research on the mentors' point of view and what they need to be able to be this ideal kind of mentor, is limited.

The fundamental nature of mentoring consists of multiple support roles and a longer duration than, for example, a traditional introduction, i.e., mentoring can be ongoing with no precise end date, since support is needed continuously (16). In addition to being a supportive relationship, mentoring can be defined as an organizational intervention to promote the growth and socialization of newly registered nurses in an organization (17). Although mentoring can be defined in various ways, most studies agree that mentoring is relational and developmental, has career and psychosocial functions, and involves phases and transitions (13). The phases can range, for example, from discovery and testing to reciprocal interactions; in the end, the mentee perceives themselves as equal to their mentors regarding their professional capability (11).

For peer review only - http://bmjopen.bmj.com/site/about/guidelines.xhtml

Dall 'Alba (18) explained this transition to a professional role in terms of becoming. Personal growth and professional development occur in stages that rely on constructive relationships and support from other professionals, e.g., mentors. Becoming a professional is more than obtaining the knowledge and skills from an education. 'Becoming' is a learning process that transforms the whole person while their professional identity is being discovered. Learning professional ways of being involves the integration of knowing, acting, and being the professional in question (18).

In summary, the benefits and implications of mentoring and the qualities of an ideal mentoring relationship are known—from the mentee's perspective. A scoping review by Aldosari et al. (19) showed a lack of qualitative research regarding the experiences and perceptions of mentors in nursing practice. The present study will help fill the knowledge gap of mentoring from the mentors' point of view. The aim of the present study is to describe registered nurses' experiences of being a mentor for newly qualified nurses.

METHODS

Design and context

A qualitative descriptive study design with individual interviews was chosen to obtain detailed descriptions of the participating mentors' experiences, in line with Brinkmann and Kvale (20). The composition and reporting of this study were guided by The Standards for Reporting Qualitative Research (SRQR) (21). This study is part of the overarching research program , a comprehensive Nordic project aiming to develop an evidence-based mentorship program to increase support for registered nurses in northern Sweden and northern Norway.

At the time of the interviews, the study participants worked as registered nurses in health care units in hospitals and municipalities in northern Sweden and northern Norway. The healthcare systems in the Nordic countries are organized similarly, although each organizational level has some degree of variation in the content and responsibilities. Hospital care is predominately confined to state and county levels, and municipalities are responsible for home care services and elderly and disability care; e.g., nursing homes (22).

Participants and sampling

Participants were selected through purposive sampling (23) to ensure inclusion of individuals with extensive knowledge of the subject matter, i.e., the experiences of being a mentor. Seeking a diverse range of experiences, we welcomed mentors with varying backgrounds, numbers of mentees, and different mentoring routines within their respective units. We observed variations in mentoring practices across units, both within and between countries. Thus, it was imperative to include registered nurses from diverse contexts, various hospital settings, and even rural areas to establish a comprehensive and representative sample for an in-depth study of mentoring.

First, we sent an email invitation to the operational managers in all units (hospital units and municipal care units) in one northern county in each country. Operational managers from thirteen Swedish and eight Norwegian units approved the invitation. Then, managers investigated interest and provided contact information to registered nurses willing to participate. The inclusion criteria were to have been a mentor to at least one newly qualified nurse, hold permanent employment of 75%–100% as a registered nurse, and to be able to communicate in Swedish or Norwegian. Lastly, we invited twenty-five registered nurses. The goal was to conduct at least seventeen interviews, in line with research on saturation in qualitative research (24). In this stage, four registered nurses in

For peer review only - http://bmjopen.bmj.com/site/about/guidelines.xhtml

For peer review only

Sweden declined the invitation, and all the invited registered nurses in Norway accepted the invitation. In total, twenty-one registered nurses participated in the study.

Data collection

The research team performed one-on-one semi-structured interviews with the participants in January–May 2021 via the digital platforms Teams or Zoom. The participants chose where they were located during the digital interview; it could be at home or at work. The participants were not reimbursed for their time; however, we had an agreement with leaders in the participating units that the participants could partake in the interviews during working hours. An interview guide was developed by members of the research team who had experience of being a mentor. The interview guide was inspired by current evidence about mentoring. Reference groups in participating municipalities and counties reviewed the interview guide prior to the interviews, and a pilot interview was performed. The pilot interview confirmed that the interview questions generated answers about experiences of mentoring showing that the interview guide was valid. After consensus was reached in the research team, interview questions were established to be open-ended with reflective elements and focused on mentoring as being and becoming a mentor. Three main questions were asked; "Can you tell me what it means to be a mentor?", "Can you tell me about what you specifically focus on as a mentor?" and "How would you like to be a mentor and develop the mentorship?". Each question had follow-up questions, for example, "What made you choose to become a mentor? ". General follow-up questions throughout the interview could be "Can you give an example of...", "Can you elaborate on…", et cetera (See Interview guide, supplementary file). The median interview time was 59 minutes. Data saturation was deemed sufficient after conducting 21 interviews of varying lengths, with the longest interview lasting 90 minutes. The interviews were perceived as spontaneous and rich in content, and the interviewer followed up on the answers to elicit more detailed narratives. Interviews were audio-recorded and transcribed verbatim. Transcriptions were anonymized and stored at a secure storage space, recordings were discarded.

Data analysis

The qualitative content analysis, following the methodological approach described by Graneheim et al. (25, 26), guided the analyses. Authors analyzed both manifest and latent content. The process began with repeated readings of the text, followed by extraction of meaning units and their condensation to capture core meanings. These units were then coded by the first author (SKH), who also sorted and grouped codes with similar content. Subthemes were created by all authors, discussing the content until consensus was reached. The subthemes were thereafter abstracted into themes, illustrated in Table 1. The analysis process was iterative, moving between text, codes, abstraction, and writing, with frequent comparisons to the original text to avoid over-interpretation. Saturation was determined based on the emergence of little or no new relevant codes in the data, coupled with the repetition of issues, as described by Hennink and Kaiser (24). A consensus on themes and results was reached through discussions among all authors.

Table 1. Examples of the abstraction process

Condensed meaning units	Codes	Sub-themes	Themes
I would like to be an example; I would like the person sitting next to me to also feel that I am a role model in attitude and care. (I.5. SWE)	Being an example and role model	Striving to be a role model	Feeling motivated in being a mentor

There are always scientific changes, and we have to be receptive to that and I think it is very important that the mentor also has the ability to be flexible. (I.9.NOR)	Flexibility - be open to new knowledge	Facing new challenges	Continuously developing the learning environment
I find it frustrating sometimes. That you don't get that time to sit down and go through things in peace. (I.3.SWE)	Lack of time leads to frustration	Struggling with work organization	Navigating obstacles and cultivating support

Ethical considerations

The Swedish Ethical Review Authority (Dnr 2020-06187) and the Norwegian Centre for Research Data (No. 148896) have approved the study. We followed the recommendations on ethical principles in human research recommendations described in the Declaration of Helsinki (27). For example, participants were informed orally and in writing about the study, that participation was voluntary, and that they were free to decline to participate at any point during the study prior to publication. Data were handled with confidentiality, i.e., no unauthorized person have access to any data material.

There are always risks involved in interviewing, due to the closeness in interview situations. In other words, there is a risk of the researcher being too close or too distant (28). Although the interview questions in this study can be regarded as non-controversial, the risk described above was considered during the interviews. In addition, there was also a risk that taking part in the interviews could increase participants' workload, and/or occupy their personal time. This risk was reduced by the previously mentioned agreement with managers that participants were able to complete the interviews during their normal working hours. The risks and the benefits of the study have been taken into consideration, and the benefits have been estimated to outweigh the risks.

Trustworthiness

Following Lincoln and Guba's evaluative criteria on establishing trustworthiness (29), credibility was established through data triangulation, as participants were selected to encompass a variety of ages and genders, as well as differences in organizations/healthcare units across participating municipalities and counties. This approach included participants with diverse experiences, thereby enhancing the credibility of this study. Investigator triangulation (i.e., peer-debriefing) reinforces the study's credibility. Multiple researchers conducted the interviews, analyzed data, and interpreted the results. Uniformity in interviews was ensured by employing the same interview guide, thereby enhancing the study's dependability. The study's context and participants are described in detail, in an effort to establish transferability. The study's transferability to an international perspective should, however, be carried out in consideration of the diversities of healthcare organizations in different countries. For ensuring confirmability, reflexivity is addressed by acknowledging that researchers involved in this study had no professional obligations to the participating units or pre-existing relationships with any registered nurses participating in the study. SKH, AK, YH, and KB have backgrounds as registered nurses and experiences in mentoring and therefore had a pre-understanding about the issues addressed in this study. They have different specialties and experiences from different fields of nursing. The research team also consist of senior researchers in public health and work environments (FN and JRV).

Patient and public involvement

For peer review only - http://bmjopen.bmj.com/site/about/guidelines.xhtml
For peer review only

Begränsad delning

Patients or public were not involved in the design or planning of the study. Reference groups in participating units had the opportunity to give their opinions on the interview guide.

RESULTS

In total, twenty-one registered nurses participated in the study, from Sweden (n=13) and Norway (n=8). The participants were of various ages, genders, and had a variety of work experiences from different geographic areas and healthcare units in each county. The median age was forty-three years (range 31–59). Participants were from hospital care units (n=15) that included surgical, medical, neurological, oncology, neonatal, and intensive care units. We also interviewed participants from primary health care and municipal care (n=6).

The results suggest that being a mentor plays a crucial role in establishing safety in complex work environments. The themes and sub-themes are illustrated in Table 2. The main theme reflects participants' experiences of creating safety for their mentees, while contributing to their own professional development, and of developing a learning environment in a complex and stressful work environment. Being a mentor was described as an unclear role that needed to be clarified and defined, however they were still motivated to be a mentor.

Some aspects promoted mentoring, such as experiencing professional development and being a role model, i.e., the first theme, “feeling motivated in being a mentor”. The second theme—“continuously developing the learning environment” —covered creating safety while facing challenges and adapting in mentoring. The third theme—"navigating obstacles and cultivating support”—summed up barriers such as lack of time and support from leaders, as well as enabling factors such as supportive colleagues.

Table 2. Overview of results

Sub-themes	Themes	Main theme
Striving to be a role model	Feeling motivated in being a mentor	Being a mentor plays a crucial role in establishing safety in complex work environments
Fostering professional development		
Facing new challenges	Continuously developing the learning environment	
Adapting mentoring based on the mentee's needs		
Struggling with work organization	Navigating obstacles and cultivating support	
Enabling support and collegiality		

The themes and sub-themes are described in more detail in the following sections and verified with quotes from the participants.

Feeling motivated in being a mentor

Participants expressed how they see it as their mission to support the mentee in becoming more secure in their professional role as a registered nurse. Participants were “striving to be a role model”; which they described as one of the most crucial parts of being a mentor. Being a role model was described as multifaceted and involved a combination of personal characteristics and professional competence. Participants also reflected on the reasons why they are mentors. “Fostering

For peer review only - http://bmjopen.bmj.com/site/about/guidelines.xhtml

professional development" meant that their motivation, interest, and goals promoted being a mentor.

Striving to be a role model was expressed in various ways by the participants; for example, they talked about how they want to show moral support and strived to be trustworthy. They also described that they strived to promote self-confidence in the mentee. The participants wanted to show competence and show that they were available as mentors. As mentors, they also aimed to show a sense of safety to support the mentee in challenging situations. They saw it as exemplary to be transparent and clear about the goals of the mentoring relationship.

The participants described various qualities they strived to have as role models. The experiences they possessed as registered nurses, including communication skills and vast knowledge was part of what made them role models. Having experience was described as valuable in one's capacity to be a mentor. According to the participants, a mentor should be competent to enable sharing of knowledge and create a trusting relationship with the mentee. Having a permissive attitude was described as necessary. Other qualities of a good mentor included sensitivity, patience, and empathy. They desired to be open and neutral as a mentor. According to the participants, a mentor should strive to be self-aware, self-reflective, and convey calmness. Being a role model could consist of different aspects; for example, being there for the mentee, being a source of professional inspiration, and being supportive. One of the participants described "being supportive" as follows:

"For me, being a mentor means I'm there to support my new colleagues, to provide a bit more neutrality so that my fellow colleagues can feel safe in talking in general terms about their experiences in their nursing roles, about health and safety, about dealing with colleagues and other professional categories – being somebody that that person can turn to". (Interview 5, SWE)

The participants expressed that they wanted to inspire the mentee to be confident and secure in standing up for their decisions. By continuous mentoring, they saw that the mentee could find the professional competence and motivation to remain in the profession. In addition to showing the mentee how to be confident, participants also wanted to show the mentee that it is permissible to not know everything. It was described as essential not to steer but to guide, a mentor is someone who listens but does not have all the answers, as expressed in the following quote:

"Being a mentor means showing you don't have to know everything, but that you can still feel secure in the knowledge that you don't know everything. […] Providing moral support, secure in your professional role to the extent that you can convey a sense of security in your professional role". (Interview 1, SWE)

Fostering professional development meant that the participants were personally interested in supporting a new colleague. Being a mentor was part of the profession; they described it as 'coming with the territory'. Participants expressed that they appreciated the positive response they got from the mentees. When the mentee was interested in learning, participants were encouraged. The participants saw it as rewarding that mentoring promoted their own professional development. Growing in the profession was described as promoting job satisfaction. Mentoring created security and joy at work for both the mentor and the mentee, according to the participants. The participants expressed feelings of excitement in being a mentor. They felt they had the capacity and talent to be suitable to be a mentor and had a lot of experience and knowledge to offer. Mentors were sometimes selected by management, which could be flattering and motivating but could also jeopardize their interest and motivation as a mentor. The goal of being a mentor was to facilitate competent colleagues and ensure the professional development of the department, and mentoring could lead to the development of a sustainable work environment. Participants described it as

For peer review only - http://bmjopen.bmj.com/site/about/guidelines.xhtml

Begränsad delning

'quality assurance'. This participant shared how professional development in mentoring could benefit patient care:

"But sharing the knowledge born of experience is precisely what's so important if others are to succeed in their work, and also to achieve the best possible patient outcomes. So, I think exchanging experience is incredibly important". (Interview 15, NOR)

Regardless of how they became a mentor, the participants described factors that guided them in their mentoring. Being a mentor contributed to their professional development. Seeing mentees thrive in the profession was described as 'rewarding' and increased participant's own competence. Participants described that their competence was made visible during mentorship. A motivational factor in being a mentor was that some participants did not have a mentor when they themselves were new nurses, and they described that they missed having a mentor. In contrast, others described the drive to give the same support they had received as new nurses. Being interested in mentoring was essential to be a successful mentor, as described by this participant:

"But then I think most people could do a good job as mentors if they have an interest in it and the desire to do it. But I think you have to have an interest in it – that's the top priority. To be able to do a good job, you have to want to do it". (Interview 6, SWE)

Continuously developing the learning environment

The participants described fast-changing healthcare, which places high demands on a learning environment that support staffs in continuous development. The participants described how new nurses, in their opinion, needed different support today than they did a few years ago. "Facing new challenges" meant that they, as mentors, needed to keep up with new and updated knowledge. The participants reflected that there was no 'best way' to be a mentor, they had to "adapt their mentoring based on the mentee's needs" and knowledge base. A mentor was thought to be essential, regardless of other introductory efforts and routines. The mentees' individual needs guided how long the mentorship lasted and the content of the mentoring.

Facing new challenges meant that as mentors, they needed to have "humility for the new", as described by the participants. Mentoring and support were needed not only in clinical situations but also in interactions with other professional categories. The participants tried to foster a permissive atmosphere, where mentees felt safe asking questions. The participants also saw a need for mentoring for all new nurses, but it could be challenging if the new nurse did not think they needed mentoring. It was also described as challenging not to 'give too much' but to encourage the mentee to develop their own abilities.

Participants saw talking about feelings as essential, and that the most challenging aspect was dealing with existential issues if the mentee, for example, had cared for a dying patient. There were challenges keeping up with the development of the current requirements placed on new nurses. The participants saw a need for a longer mentoring period than simply an introduction. They had to be receptive to new knowledge, as described by this participant:

"You feel you keep your wits about you when you're a mentor, that you constantly have to go on learning new things. And not least, you have to be sure that what you're teaching is correct and up to date on a professional level". (Interview 8, NOR)

Adapting mentoring based on mentee's needs meant that mentoring could vary based on the mentee's previous experiences. With a confident mentee, the mentor could be less 'strict'. However,

For peer review only - http://bmjopen.bmj.com/site/about/guidelines.xhtml

with an insecure mentee, the mentor had to be more hands-on, according to the participants, and guide the mentee more regularly. Mentoring could be adapted to quick and irregular check-ins, as described by this participant:

"Perhaps dealing with mentoring "on the hoof", so to speak, there and then, but then maybe also being able to sit down and give it time. You adapt to the situation". (Interview 17, SWE)

Several participants described a matching process for the mentee–mentor relationship, where they saw how personal chemistry affected the mentoring. The participants emphasized they needed to know the mentee to plan the mentorship, not only the mentee's previous work experience, but also family life, other obligations outside working hours, etc.

A mentoring conversation stimulates reflection and consideration, according to the participants. The participants tried to focus on the positive aspects of the mentee's development. The ambition was to let the mentee control what was addressed in the discussions, based on the mentees' individual needs. The participants encouraged the mentee to prepare for the mentoring conversation. To be a mentor was also described by the participants as to guide in conflicts, for example if there was a disagreement with a colleague. To continuously follow up on challenges in the job.

The mentoring conversations were based on confidentiality to enable openness. The participants expressed that the aim was to meet one another unconditionally. They thought it was better with freer frames and flexibility around the conversation. It could be anywhere, based on the conditions in the workplace, if it were in a trustworthy environment. For example, mentoring could be adapted into a short and spontaneous reflection after a work shift. Mentoring can develop the mentees' professional skills without resorting to criticism, according to the participants.

"But it depends entirely on who I'm mentoring, [the mentee] is the one who should decide what we're going to talk about". (Interview 2, SWE)

Navigating obstacles and cultivating support

The participants expressed experiences of navigating obstacles in the work environment, that could hinder being a mentor fully. They described "struggles with work organizations", and they experienced pressure from leaders and colleagues. The participants also reflected on factors that cultivated mentorship in a supportive environment. They reported that mentoring was enabled if leaders were attentive, and the organization was well-functioning. "Enabling support and collegiality" was also expressed as a necessary part of a supportive environment. Participants also claimed that having structure and predictability in the working day helped them in planning mentoring activities, and that structure and predictability were essential elements of cultivating a supportive environment.

Struggling with work organization meant that participants expressed obstacles related to and frustration with how leaders and management handled mentorship. Responsiveness and sensitivity of managers were lacking, according to the participants. Participants had experienced that they were handed responsibility but not power to influence. Participants requested clarification regarding what mentorship was supposed to be, to be able to navigate the mentor role. For example, this participant expressed thoughts of ending their mentorship due to unclear definitions of the role:

"Given that [the mentoring role] has been a bit woolly, I was almost starting to think I should give up mentoring because I have plenty of other things going on at work. [...] Maybe things are getting a bit

For peer review only - http://bmjopen.bmj.com/site/about/guidelines.xhtml

Begränsad delning

much, and if I had to turn anything down, then the mentoring would be the first thing to go". (Interview 7, SWE)

They said it was not beneficial for the work environment to 'be forced' to be a mentor. Participants also perceived mentoring to be a non-priority in the eyes of management. Mentoring was often de-prioritized for other tasks in the workplace.

It was challenging to be a mentor due to insufficient training in, for example, conversational techniques and motivational conversations. They expressed that "mentoring in mentoring" and contact with other mentors were lacking. The will was there—but education and development in mentoring needed to be improved to be able to cultivate a supportive environment. The participants described experiences of staff shortages and high staff turnover that led to obstacles in mentoring. For example, participants had experiences of simultaneously mentoring several new nurses, which could lead to difficulties in navigating the time management and priorities.

They expressed a need for better planning and structure within the organization. It was described as necessary with sensitivity between the workgroup and the mentee. However, there needed to be a more precise division of responsibilities for follow-up in the mentoring. They experienced pressure from colleagues and felt responsible for the mentee and their skills. It was described as challenging to mentor someone whom colleagues saw as an insecure mentee.

Difficulties in planning, such as scheduling factors, could be obstacles for mentoring; there needed to be more time set aside for mentoring. The lack of time led to frustration, and not having set aside time made learning difficult, according to the participants. They needed help preparing. For example, they prepared for mentoring outside working hours. They streamlined mentoring due to lack of time, which led to insufficient development in the mentee. The participants experienced difficulties in navigating mentoring activities during periods of high workload. However, they saw a greater need for mentoring during these periods. This frustration is expressed by one of the participants in this quote:

"The management must take the time to follow up. No progress will be made unless they do. But they often feel there's no time for it, so it's not a priority. So, it's there, but it grinds to a halt". (Interview 14, NOR)

Enabling support and collegiality meant that a structured environment was essential for being a mentor. Receiving support from team leaders reduced the obstacles and improved the possibilities to enable mentorship. Good management and leadership led to personalized mentoring, according to the participants. When leaders made it possible for them to navigate in both informal and formal structures for mentorship, this was an example of an attentive leader. This is expressed in the following quote:

"I also have to make it clear that we have a good manager who pays attention to everyone and understands everyone. She's also really good at keeping things on track. I've often felt that as our manager, she's the glue that bonds the group together". (Interview 12, NOR)

The participants stated that flexibility in scheduling and planning was a prerequisite for being a successful mentor. According to the participants, having time allocated for both mentoring and patient-related work was a prerequisite for cultivating support. Having time for reflection (less workload) also promoted being a mentor. The participants described that having a mentee could reduce but also increase the workload. They described alternating mentorship as preferable, having some space between mentorships and replacing each other during absences.

For peer review only - http://bmjopen.bmj.com/site/about/guidelines.xhtml

The participants expressed that it was important to be loyal to one another, even though they were all different and could do things differently. It was described as essential to receive support from other mentors (for example, to talk about obstacles and "difficult cases"). Sharing the responsibility and supporting other mentors and colleagues were considered cultivating. Participants expressed a will to build a value base for mentoring, create a mentorship structure, navigate a plan together. They gave examples of mentor groups where they planned new and reflected on previous mentorships. They expressed that they needed more regular meetings to exchange experiences.

They also shared how everyone in the workgroup took part in the mentoring, but only some had the title 'mentor'. Discussions were requested between their colleagues in the workgroup on frameworks and responsibilities regarding mentorship. Participants expressed the importance of taking care of one another. According to the participants, everyone in the workgroup had to be prepared and welcoming to the mentee, and the social work environment was essential for the mentees' learning. The workplace had to be inclusive and responsive, according to the participants. Participants also expressed that supportive leaders and a supportive organization were essential for them continuing to be a mentor. Attitudes and organizational culture were described as vital components in successful mentoring:

"But just like here, people need a management team that does the same thing. I can't be the only one, I rely on us having the same ideas about [mentoring]. It's really important". (Interview 13, NOR)

DISCUSSION

The principal findings are that the complexity of the mentor role was evident in the registered nurses' narratives, and they strived to establish safety for mentees in complex work environments. Results show that the mentor role lacked clarity and entailed positive as well as negative experiences. For example, participants talked about being motivated, continuously developing the learning environment, and navigating obstacles and cultivating support.

Feeling motivated being in the role of a mentor

The results of the study show that being a mentor includes emphasis on creating trusting relationships with their mentees and being able to share knowledge with one's mentees. These results are in accordance with previous studies that concluded that mentoring success depends on the quality of the mentoring relationships, i.e., the personal connection between the mentor and the mentee (30, 31, 32). Also, according to Dall 'Alba (18), people cannot become professionals by themselves; they can fully develop through contact and integration with others. Participants in our study strived to be a role model and offer support to their new colleagues, while at the same time develop in their own new professional identity of being a mentor.

In addition to supporting their mentees' professional development, being a mentor was also described as beneficial for the participants' own personal and professional development, as confirmed in previous research (31). The participants in our study expressed that being a role model, being trustworthy, and having competence and experience as a nurse were prerequisites for mentoring. This confirms previous descriptions of an ideal mentor from the mentees' point of view that emphasized similar characteristics (12, 13).

Continuously developing the learning environment

The present study adds new perspectives to previous studies regarding mentorship. For example, a systematic review by Kakyo et al. (31) concluded that the content of mentoring is vital for it to work.

For peer review only - http://bmjopen.bmj.com/site/about/guidelines.xhtml

Begränsad delning

In contrast, our results showed that it was important to have freer frames and adapt mentoring based on mentees' needs. Our results showed that content was not the most important aspect of mentorship; the participants in our study emphasized more about *how* and *why* mentoring was performed in their narratives. To develop a learning environment, a need to build a value base for mentoring and create a mentorship structure together with other mentors and colleagues was shown.

The experiences of developing the learning environment and being a role model can be compared to how Dall 'Alba (18) describes "learning to become a professional" as an interplay between "what I am and what I can become". This means inheriting a world of other professionals, as well as inventing one's own world as a professional. Our study shows similar findings. Being a mentor meant having a sense of security in their own professional role and instilling self-confidence in their new colleagues, so that they, in turn, can build their own professional identities. This is confirmative to Yip et al. (33) who showed in their study that support from senior colleagues gave new nurses hope, and facilitated their self-transformative process in their professional development as registered nurses.

Other than getting to know their mentee and adapting to their personal learning needs, participants in the present study also expressed challenges in keeping up with the rapid development of new knowledge. A meta-synthesis by Mlambo et al. (34) supports these findings by concluding that organizations should adequately make continuing professional development accessible for registered nurses to keep the staff up to date.

Navigating obstacles and cultivating support

The results of the present study show experiences of staff shortage and time shortage, which hindered participants' ability to perform mentoring activities and participate fully in being a mentor. The participants expressed a need for education, training, meeting other mentors, and the importance of relationships, i.e., 'not doing it alone'. These results are confirmed by previous research that showed how education, training, and time allocated for the mentor to interact with the mentee are prerequisites for organizations to benefit from mentoring (31). Other studies showed that workload management and scheduling influence how mentoring can be conducted and showed that nurse managers expressed similar barriers to mentoring, such as competing priorities for time, and lack of training (30, 35).

Understaffing is related to emotional exhaustion and lower well-being in the workgroup. The higher workload from understaffing also leads to role ambiguity; i.e., staff being unclear about their roles (36). Results in our study confirms findings of role ambiguity, as participants described thoughts of ending the mentorship due to unclear definitions of the responsibility it entailed and their role as mentors. In addition, participants in the present study also described other challenges; needing structure, i.e., clear leadership and direction, to optimize the mentoring. These findings can be interpreted as participants experiencing inequity, that their input in mentorship is unequal to the outcomes they receive, as described in *equity theory* by Adams (37). Inequity can, according to Adams (37) lead to frustration, job-dissatisfaction, and a person leaving their position.

The results of our study both confirmed and expanded prior research. Even so, mentoring is still not a part of everyday routines in all healthcare units. The importance and benefits of mentoring are known, but it is still not facilitated sufficiently. According to Dall 'Alba (18), a person's possibilities to act and be are constrained by specific situations, as well as their history, traditions, and conditions. The current situation in health care is not ideal for fully developing mentoring. There is a vicious cycle, where the high turnover of registered nurses leads to difficulties in implementing mentoring that contributes to difficulties retaining registered nurses. A recent report from the International

For peer review only - http://bmjopen.bmj.com/site/about/guidelines.xhtml

Centre on Nurse Migration (ICNM) showed that the demand for RNs is on the rise in several countries and estimated that there will be a need for 13 million RNs globally, in the next decade (38). Other reports on the global shortage of registered nurses (39, 40) have concluded that organizations must create positive work environments that maximize registered nurses' health, safety, and well-being, and improve or sustain their motivation to prevent further shortages. Registered nurses are attracted to work and remain working due to, for example, opportunities to develop professionally, gain autonomy, and participate in decision-making (39, 40).

Moreover, the results of the present study show the frustration evident in the participating registered nurses' narratives. They did not feel valued by the organization and experienced inequity. Therefore, they could not thoroughly add value to their mentorships or to the organization in return. A systematic review of interventions that promote registered nurses' retention by Lartey et al. (41) concluded that most interventions have focused on retaining newly qualified nurses. However, keeping experienced registered nurses in the profession is essential for providing high-quality care, i.e., effective, safe, evidence-based, and person-centered healthcare to meet the evolving needs of patients (42). Organizations need to invest in experienced registered nurses to encourage them to remain in the profession, to provide high-quality care, and as mentors for new nurses (6). Therefore, listening to the registered nurses' experiences and giving them opportunities to develop professionally and participate in decision-making about mentoring are essential factors in keeping them in the profession and for their well-being.

For peer review only

Strengths and weaknesses of the study

Having a variety of backgrounds and understandings in the research team broadened the discussions and proved an advantage in our ability to understand the data. A strength is also that we described our pre-understanding, and our diversity of backgrounds that might prevent bias and achieve consensus through co-creation; as different researchers in a study can interpret the data differently (25). The researchers speak Swedish and Norwegian, and our variety in native languages was a strength, e.g., we could validate the transcripts and analysis by ensuring that nothing got lost in translation. A weakness of the study can be that the first author (SKH) analyzed the interviews but did not conduct any of the interviews. It can be a disadvantage where part of the latent content, such as nonverbal behaviors and interactions, can be missed in the analysis. However, all other authors performed several of the interviews and was part of the analyses process.

Clinical implications

This study brings a more profound understanding of registered nurses' perspectives of being a mentor. This study offers evidence as to how mentoring should be facilitated, thus contributing to highly competent registered nurses and safer patient care.

Suggestions for further research

More research is required to fully explore and implement mentoring as a concept. In addition, more research is needed to develop, test, and evaluate evidence-based mentorship programs in healthcare units and to create sustainable mentoring solutions.

CONCLUSIONS

Being a mentor is a complicated role for registered nurses. The mentoring role is beneficial, i.e., positive and rewarding, if facilitated effectively. The role and responsibilities in the mentoring relationship need to be fully established and defined. A structured organization is essential to

For peer review only - http://bmjopen.bmj.com/site/about/guidelines.xhtml

promote mentoring, as is taking experienced registered nurses' views into consideration. The structure of mentoring in nursing should be designed and facilitated in co-creation with experienced registered nurses as mentors.

DECLARATIONS

Ethics approval and consent: The Swedish Ethical Review Authority (Dnr 2020-06187) and the Norwegian Centre for Research Data (No. 148896) have approved the study. Written informed consent for participation and publication was obtained.

Data sharing statement: No data are available.

Competing interests: None declared.

Funding: This work was supported by Nordland County [20/1879-14].

Authors contributions:
SKH: Data analysis, Writing – original draft, Writing – review & editing.
AK: Data collection, Validation of analysis, Writing – review & editing.
YH: Project administration, Supervision, Data collection, Writing – review & editing.
FN: Supervision, Writing – review & editing.
JRV: Data collection, Writing – review & editing.
KB: Project administration, Supervision, Data collection, Validation of analysis, Writing – review & editing.

Acknowledgements: Our deepest gratitude to all the participating registered nurses for generously sharing their narratives. We want to thank Johan Åhlin and Jonas Carlström for collecting data. We also want to thank Nord University and Umeå University for the support they provided, making it possible to carry out the study.

REFERENCES

1. Pham TTL, Teng CI, Friesner D, Li K, Wu WE, Liao YN, et al. The impact of mentor–mentee rapport on nurses' professional turnover intention: Perspectives of social capital theory and social cognitive career theory. Journal of Clinical Nursing. 2019;28(13-14):2669-80.
2. Schroyer CC, Zellers R, Abraham S. Increasing Registered Nurse Retention Using Mentors in Critical Care Services. The Health Care Manager. 2020;39(2):85-99.
3. Mijares AH, Radovich P. Structured Mentorship and the Nursing Clinical Ladder. Clinical Nurse Specialist. 2020;34(6):276-81.
4. Coventry TH, Maslin-Prothero SE, Smith G. Organizational impact of nurse supply and workload on nurses continuing professional development opportunities: an integrative review. Journal of Advanced Nursing. 2015;71(12):2715-27.
5. Edwards D, Hawker C, Carrier J, Rees C. A systematic review of the effectiveness of strategies and interventions to improve the transition from student to newly qualified nurse. International Journal of Nursing Studies. 2015;52(7):1254-68.
6. Jangland E, Gunningberg L, Nyholm L. A mentoring programme to meet newly graduated nurses' needs and give senior nurses a new career opportunity: A multiple-case study. Nurse Education in Practice. 2021;57:103233-.
7. Cambridge Dictionary 2022 [Available from: https://dictionary.cambridge.org/dictionary/english/mentoring.

For peer review only - http://bmjopen.bmj.com/site/about/guidelines.xhtml

8. Olorunfemi O. Mentoring in Nursing: A Concept Analysis. International Journal of Caring Sciences 2019;12(1):142-8.
9. Zhang Y, Qian Y, Wu J, Wen F, Zhang Y. The effectiveness and implementation of mentoring program for newly graduated nurses: A systematic review. Nurse Education Today. 2016;37:136-44.
10. Hoover J, Koon AD, Rosser EN, Rao KD. Mentoring the working nurse: a scoping review. Human Resources for Health. 2020;18(1):52-.
11. Hale RL, Phillips CA. Mentoring up: A grounded theory of nurse-to-nurse mentoring. Journal of Clinical Nursing. 2019;28(1-2):159-72.
12. Chen CM, Lou MF. The effectiveness and application of mentorship programmes for recently registered nurses: a systematic review. Journal of Nursing Management. 2014;22(4):433-42.
13. Mullen CA, Klimaitis CC. Defining mentoring: a literature review of issues, types, and applications. Annals of the New York Academy of Sciences. 2021;1483(1):19-35.
14. Gularte-Rinaldo J, Baumgardner R, Tilton T, Brailoff V. Mentorship ReSPeCT Study: A Nurse Mentorship Program's Impact on Transition to Practice and Decision to Remain in Nursing for Newly Graduated Nurses. Nurse Leader 2023;21(2):262-7.
15. Hampton KB, Smeltzer SC, Ross JG. Evaluating the transition from nursing student to practicing nurse: An integrative review. Journal of Professional Nursing. 2020;36(6):551-9.
16. Hansen J. Results from the national council of state boards of nursing's transition-to-practice study, part 2. Journal for Nurses in Professional Development 2015;31(4):240-1.
17. Krofft K, Stuart W. Implementing a Mentorship Program for New Nurses During a Pandemic. Nursing Administration Quarterly. 2021;45(2):152-8.
18. Dall'Alba G. Learning Professional Ways of Being: Ambiguities of becoming. Educational Philosophy and Theory. 2013;41(1):34-45.
19. Aldosari N, Pryjmachuk S, Cooke H. Newly qualified nurses' transition from learning to doing: A scoping review. International Journal of Nursing Studies. 2021;113.
20. Brinkmann S, Kvale S. Doing interviews. 2nd edition. ed. London: SAGE Publications Ltd; 2018.
21. O'Brien BC, Harris IB, Beckman TJ, Reed DA, Cook DA. Standards for Reporting Qualitative Research: A Synthesis of Recommendations. Academic Medicine. 2014;89(9):1245-51.
22. The Nordic Medico-Statistical Committee and The Nordic Social Statistical Committee; Nordic Health & Welfare Statistics: The Nordic Medico-Statistical Committee and The Nordic Social Statistical Committee; 2022 [Available from: https://nhwstat.org/health/organization-health-services.
23. Creswell JW. Qualitative inquiry and research design: choosing among five approaches. 3., [updated] ed. Thousand Oaks: SAGE Publications; 2013.
24. Hennink M, Kaiser BN. Sample sizes for saturation in qualitative research: A systematic review of empirical tests. Social Science & Medicine. 2022;292:114523.
25. Graneheim UH, Lindgren BM, Lundman B. Methodological challenges in qualitative content analysis: A discussion paper. Nurse Education Today. 2017;56:29-34.
26. Graneheim UH, Lundman B. Qualitative content analysis in nursing research: concepts, procedures and measures to achieve trustworthiness. Nurse Education Today. 2004;24(2):105-12.
27. World Medical Association Declaration of Helsinki: Ethical Principles for Medical Research Involving Human Subjects. Journal of the American Medical Association. 2013;310(20):2191-4.
28. Haahr A, Norlyk A, Hall EO. Ethical challenges embedded in qualitative research interviews with close relatives. Nursing Ethics. 2014;21(1):6-15.
29. Lincoln YS, Guba EG, Pilotta JJ. Naturalistic inquiry. International Journal of Intercultural Relations. 1985;9(4):438-9.
30. Devey Burry R, Stacey D, Backman C, Donia MBL, Lalonde M. Exploring pairing of new graduate nurses with mentors: An interpretive descriptive study. Journal of Clinical Nursing. 2020;29(15-16):2897-906.

For peer review only - http://bmjopen.bmj.com/site/about/guidelines.xhtml

31. Kakyo TA, Xiao LD, Chamberlain D. Benefits and challenges for hospital nurses engaged in formal mentoring programs: A systematic integrated review. International Nursing Review. 2022;69(2):229-38.
32. Lin J, Chew YR, Toh YP, Radha Krishna LK. Mentoring in Nursing: An Integrative Review of Commentaries, Editorials, and Perspectives Papers. Nurse Educator. 2018;43(1):E1-E5.
33. Yip Y-C, Yip K-H, Tsui W-K. The transformational experience of junior nurses resulting from providing care to COVID-19 patients: from facing hurdles to achieving psychological growth. International Journal of Environmental Research and Public Health. 2021;18(14):7383.
34. Mlambo M, Silén C, McGrath C. Lifelong learning and nurses' continuing professional development, a metasynthesis of the literature. BMC Nursing. 2021;20(1):62-.
35. Merga MK, Hays A-M, Coventry T. Nurse managers' perceptions of barriers to the mentoring of early career nurses. Mentoring & Tutoring. 2020;28(1):60-77.
36. Hudson CK, Shen W. Consequences of work group manpower and expertise understaffing: A multilevel approach. Journal of Occupational Health Psychology. 2018;23(1):85.
37. Adams JS. Inequity in social exchange. Advances in Experimental Social Psychology. 1965;2(4):267-99.
38. Buchan J, Catton H, Shaffer F. Sustain and Retain in 2022 and Beyond: The global nursing workforce and the COVID-19 pandemic. International Centre on Nurse Migration. 2022.
39. Buchan J. Global nursing shortages. BMJ. 2002;324(7340):751-2.
40. Drennan VM, Ross F. Global nurse shortages: the facts, the impact and action for change. British Medical Bulletin. 2019;130(1):25-37.
41. Lartey S, Cummings G, Profetto-McGrath J. Interventions that promote retention of experienced registered nurses in health care settings: a systematic review. Journal of Nursing Management. 2014;22(8):1027-41.
42. World Health Organization. Handbook for national quality policy and strategy: a practical approach for developing policy and strategy to improve quality of care. 2018 16 April 2018. Report No.: 9789241565561.

For peer review only

Supplementary file

Interview guide, individual interviews with mentors (translated from Swedish by the first author)

Information:
• Brief oral presentation of the project
• Mentors introduce themselves
• Anonymity and confidentiality
• Information about audio recording and data storage
• Consent to participate in the study
• Test audio recording. Start recording and interview

Interview questions:

1. **Can you tell me what it means to be a mentor?**
   - What made you choose to become a mentor?
   - If you were appointed as a mentor, why do you think you were chosen?
   - What drives you as a mentor? What is your driving force?

2. **Can you tell me about what you specifically focus on as a mentor?**
   - Can you tell me about the organization of mentorship in your workplace?
   - Can you tell me what promotes mentoring in your workplace and what can prevent (inhibit) mentoring in the workplace?
   - How does your work environment contribute to you being able to perform mentoring?
   - Tell me about the preparations to welcome a new colleague

3. **How would you like to be a mentor and develop the mentorship?**
   - Can you tell me what it means to go from being a "helpful colleague" to being a mentor?
   - If you had a mentor who was your role model, can you describe what qualities you liked in his/her mentoring?
   - Tell me how you would develop mentorship in your unit

Final question: Is there anything else you would like to say about mentorship that you don't think I have asked?

General follow-up questions (questions that are adapted during the interview):
- Can you tell me more about/elaborate on ...
- Can you give an example ...
- Do you think that...

For peer review only

Standards for Reporting Qualitative Research (SRQR)*

http://www.equator-network.org/reporting-guidelines/srqr/

Title and abstract

	Page/line no(s).
**Title** - Concise description of the nature and topic of the study Identifying the study as qualitative or indicating the approach (e.g., ethnography, grounded theory) or data collection methods (e.g., interview, focus group) is recommended	1
**Abstract** - Summary of key elements of the study using the abstract format of the intended publication; typically includes background, purpose, methods, results, and conclusions	1

Introduction

**Problem formulation** - Description and significance of the problem/phenomenon studied; review of relevant theory and empirical work; problem statement	2-3
**Purpose or research question** - Purpose of the study and specific objectives or questions	3

Methods

**Qualitative approach and research paradigm** - Qualitative approach (e.g., ethnography, grounded theory, case study, phenomenology, narrative research) and guiding theory if appropriate; identifying the research paradigm (e.g., postpositivist, constructivist/ interpretivist) is also recommended; rationale**	3
**Researcher characteristics and reflexivity** - Researchers' characteristics that may influence the research, including personal attributes, qualifications/experience, relationship with participants, assumptions, and/or presuppositions; potential or actual interaction between researchers' characteristics and the research questions, approach, methods, results, and/or transferability	5
**Context** - Setting/site and salient contextual factors; rationale**	3
**Sampling strategy** - How and why research participants, documents, or events were selected; criteria for deciding when no further sampling was necessary (e.g., sampling saturation); rationale**	3-4
**Ethical issues pertaining to human subjects** - Documentation of approval by an appropriate ethics review board and participant consent, or explanation for lack thereof; other confidentiality and data security issues	5
**Data collection methods** - Types of data collected; details of data collection procedures including (as appropriate) start and stop dates of data collection and analysis, iterative process, triangulation of sources/methods, and modification of procedures in response to evolving study findings; rationale**	4

For peer review only - http://bmjopen.bmj.com/site/about/guidelines.xhtml

For peer review only

**Data collection instruments and technologies** - Description of instruments (e.g., interview guides, questionnaires) and devices (e.g., audio recorders) used for data collection; if/how the instrument(s) changed over the course of the study	4
**Units of study** - Number and relevant characteristics of participants, documents, or events included in the study; level of participation (could be reported in results)	3-4 and 6
**Data processing** - Methods for processing data prior to and during analysis, including transcription, data entry, data management and security, verification of data integrity, data coding, and anonymization/de-identification of excerpts	4
**Data analysis** - Process by which inferences, themes, etc., were identified and developed, including the researchers involved in data analysis; usually references a specific paradigm or approach; rationale**	4
**Techniques to enhance trustworthiness** - Techniques to enhance trustworthiness and credibility of data analysis (e.g., member checking, audit trail, triangulation); rationale**	5

Results/findings

**Synthesis and interpretation** - Main findings (e.g., interpretations, inferences, and themes); might include development of a theory or model, or integration with prior research or theory	6-11
**Links to empirical data** - Evidence (e.g., quotes, field notes, text excerpts, photographs) to substantiate analytic findings	6-11

Discussion

**Integration with prior work, implications, transferability, and contribution(s) to the field -** Short summary of main findings; explanation of how findings and conclusions connect to, support, elaborate on, or challenge conclusions of earlier scholarship; discussion of scope of application/generalizability; identification of unique contribution(s) to scholarship in a discipline or field	11-13
**Limitations** - Trustworthiness and limitations of findings	13

Other

**Conflicts of interest** - Potential sources of influence or perceived influence on study conduct and conclusions; how these were managed	14
**Funding** - Sources of funding and other support; role of funders in data collection, interpretation, and reporting	14

*The authors created the SRQR by searching the literature to identify guidelines, reporting standards, and critical appraisal criteria for qualitative research; reviewing the reference lists of retrieved sources; and contacting experts to gain feedback. The SRQR aims to improve the transparency of all aspects of qualitative research by providing clear standards for reporting qualitative research.

For peer review only

**The rationale should briefly discuss the justification for choosing that theory, approach, method, or technique rather than other options available, the assumptions and limitations implicit in those choices, and how those choices influence study conclusions and transferability. As appropriate, the rationale for several items might be discussed together.

Reference:

O'Brien BC, Harris IB, Beckman TJ, Reed DA, Cook DA. **Standards for reporting qualitative research: a synthesis of recommendations.** *Academic Medicine*, Vol. 89, No. 9 / Sept 2014 DOI: 10.1097/ACM.0000000000000388

For peer review only
